# Severity of Respiratory Syncytial Virus vs COVID-19 and Influenza Among Hospitalized US Adults

**DOI:** 10.1001/jamanetworkopen.2024.4954

**Published:** 2024-04-04

**Authors:** Diya Surie, Katharine A. Yuengling, Jennifer DeCuir, Yuwei Zhu, Adam S. Lauring, Manjusha Gaglani, Shekhar Ghamande, Ithan D. Peltan, Samuel M. Brown, Adit A. Ginde, Amanda Martinez, Nicholas M. Mohr, Kevin W. Gibbs, David N. Hager, Harith Ali, Matthew E. Prekker, Michelle N. Gong, Amira Mohamed, Nicholas J. Johnson, Vasisht Srinivasan, Jay S. Steingrub, Aleda M. Leis, Akram Khan, Catherine L. Hough, William S. Bender, Abhijit Duggal, Emily E. Bendall, Jennifer G. Wilson, Nida Qadir, Steven Y. Chang, Christopher Mallow, Jennie H. Kwon, Matthew C. Exline, Nathan I. Shapiro, Cristie Columbus, Ivana A. Vaughn, Mayur Ramesh, Jarrod M. Mosier, Basmah Safdar, Jonathan D. Casey, H. Keipp Talbot, Todd W. Rice, Natasha Halasa, James D. Chappell, Carlos G. Grijalva, Adrienne Baughman, Kelsey N. Womack, Sydney A. Swan, Cassandra A. Johnson, Cara T. Lwin, Nathaniel M. Lewis, Sascha Ellington, Meredith L. McMorrow, Emily T. Martin, Wesley H. Self

**Affiliations:** 1 Coronavirus and Other Respiratory Viruses Division, National Center for Immunization and Respiratory Diseases, Centers for Disease Control and Prevention, Atlanta, Georgia; 2 Department of Biostatistics, Vanderbilt University Medical Center, Nashville, Tennessee; 3 Department of Internal Medicine, University of Michigan, Ann Arbor; 4 Department of Microbiology and Immunology, University of Michigan, Ann Arbor; 5 Baylor Scott & White Health, Temple, Texas; 6 Texas A&M University College of Medicine, Temple; 7 Baylor College of Medicine, Temple, Texas; 8 Department of Medicine, Intermountain Medical Center, Murray, Utah and University of Utah, Salt Lake City; 9 Department of Emergency Medicine, University of Colorado School of Medicine, Aurora; 10 University of Iowa, Iowa City; 11 Department of Medicine, Wake Forest School of Medicine, Winston-Salem, North Carolina; 12 Department of Medicine, Johns Hopkins University School of Medicine, Baltimore, Maryland; 13 Department of Emergency Medicine, Hennepin County Medical Center, Minneapolis, Minnesota; 14 Department of Medicine, Montefiore Medical Center, Albert Einstein College of Medicine, Bronx, New York; 15 Department of Emergency Medicine, Division of Pulmonary, Critical Care and Sleep Medicine, University of Washington, Seattle; 16 Department of Emergency Medicine, University of Washington, Seattle; 17 Department of Medicine, Baystate Medical Center, Springfield, Massachusetts; 18 School of Public Health, University of Michigan, Ann Arbor; 19 Department of Medicine, Oregon Health and Sciences University, Portland; 20 Department of Medicine, Emory University, Atlanta, Georgia; 21 Department of Medicine, Cleveland Clinic, Cleveland, Ohio; 22 Department of Emergency Medicine, Stanford University School of Medicine, Stanford, California; 23 Department of Medicine, University of California, Los Angeles; 24 Department of Medicine, University of Miami, Miami, Florida; 25 Department of Medicine, Washington University in St Louis, St Louis, Missouri; 26 Department of Medicine, The Ohio State University, Columbus; 27 Department of Emergency Medicine, Beth Israel Deaconess Medical Center, Boston, Massachusetts; 28 Baylor Scott &White Health, Dallas, Texas; 29 Texas A&M University College of Medicine, Dallas; 30 Department of Public Health Sciences, Henry Ford Health, Detroit, Michigan; 31 Division of Infectious Diseases, Henry Ford Health, Detroit, Michigan; 32 Department of Emergency Medicine, University of Arizona, Tucson; 33 Yale University School of Medicine, New Haven, Connecticut; 34 Department of Medicine, Vanderbilt University Medical Center, Nashville, Tennessee; 35 Department of Health Policy, Vanderbilt University Medical Center, Nashville, Tennessee; 36 Department of Pediatrics, Vanderbilt University Medical Center, Nashville, Tennessee; 37 Department of Emergency Medicine, Vanderbilt University Medical Center, Nashville, Tennessee; 38 Vanderbilt Institute for Clinical and Translational Research, Vanderbilt University Medical Center, Nashville, Tennessee; 39 Influenza Division, National Center for Immunization and Respiratory Diseases, Centers for Disease Control and Prevention, Atlanta, Georgia

## Abstract

**Importance:**

On June 21, 2023, the Centers for Disease Control and Prevention recommended the first respiratory syncytial virus (RSV) vaccines for adults aged 60 years and older using shared clinical decision-making. Understanding the severity of RSV disease in adults can help guide this clinical decision-making.

**Objective:**

To describe disease severity among adults hospitalized with RSV and compare it with the severity of COVID-19 and influenza disease by vaccination status.

**Design, Setting, and Participants:**

In this cohort study, adults aged 18 years and older admitted to the hospital with acute respiratory illness and laboratory-confirmed RSV, SARS-CoV-2, or influenza infection were prospectively enrolled from 25 hospitals in 20 US states from February 1, 2022, to May 31, 2023. Clinical data during each patient’s hospitalization were collected using standardized forms. Data were analyzed from August to October 2023.

**Exposures:**

RSV, SARS-CoV-2, or influenza infection.

**Main Outcomes and Measures:**

Using multivariable logistic regression, severity of RSV disease was compared with COVID-19 and influenza severity, by COVID-19 and influenza vaccination status, for a range of clinical outcomes, including the composite of invasive mechanical ventilation (IMV) and in-hospital death.

**Results:**

Of 7998 adults (median [IQR] age, 67 [54-78] years; 4047 [50.6%] female) included, 484 (6.1%) were hospitalized with RSV, 6422 (80.3%) were hospitalized with COVID-19, and 1092 (13.7%) were hospitalized with influenza. Among patients with RSV, 58 (12.0%) experienced IMV or death, compared with 201 of 1422 unvaccinated patients with COVID-19 (14.1%) and 458 of 5000 vaccinated patients with COVID-19 (9.2%), as well as 72 of 699 unvaccinated patients with influenza (10.3%) and 20 of 393 vaccinated patients with influenza (5.1%). In adjusted analyses, the odds of IMV or in-hospital death were not significantly different among patients hospitalized with RSV and unvaccinated patients hospitalized with COVID-19 (adjusted odds ratio [aOR], 0.82; 95% CI, 0.59-1.13; *P* = .22) or influenza (aOR, 1.20; 95% CI, 0.82-1.76; *P* = .35); however, the odds of IMV or death were significantly higher among patients hospitalized with RSV compared with vaccinated patients hospitalized with COVID-19 (aOR, 1.38; 95% CI, 1.02-1.86; *P* = .03) or influenza disease (aOR, 2.81; 95% CI, 1.62-4.86; *P* < .001).

**Conclusions and Relevance:**

Among adults hospitalized in this US cohort during the 16 months before the first RSV vaccine recommendations, RSV disease was less common but similar in severity compared with COVID-19 or influenza disease among unvaccinated patients and more severe than COVID-19 or influenza disease among vaccinated patients for the most serious outcomes of IMV or death.

## Key Points

**Question:**

How does disease severity of respiratory syncytial virus (RSV) compare with COVID-19 and influenza among adults hospitalized with these infections?

**Findings:**

In this cohort study of 7998 hospitalized adults aged 18 years and older in 20 US states during February 2022 to May 2023, RSV disease severity was similar to unvaccinated patients hospitalized with COVID-19 or influenza, but substantially more severe than vaccinated patients hospitalized with COVID-19 or influenza disease.

**Meaning:**

These findings suggest that before RSV vaccine introduction in the US, RSV disease was at least as severe as COVID-19 or influenza among unvaccinated patients and more severe than COVID-19 or influenza among vaccinated patients hospitalized with those diseases.

## Teaser

This cohort study examines disease severity among adults hospitalized with respiratory syncytial virus (RSV) and compares it with the severity of COVID-19 and influenza by vaccination status.

## Introduction

Respiratory syncytial virus (RSV) is increasingly recognized as an important cause of severe respiratory disease in adults. An estimated 60 000 to 160 000 RSV-associated hospitalizations and 6000 to 10 000 deaths occur each year among adults aged 65 years and older in the US.^1,2,3,4,5,6^ In May 2023, the US Food and Drug Administration approved 2 RSV vaccines for use in adults aged 60 years and older.^7^ On June 21, 2023, the Centers for Disease Control and Prevention recommended these new RSV vaccines for adults aged 60 years and older with decisions on whether to be vaccinated based on shared clinical decision-making between patient and health care practitioner.^7^ Understanding the severity of RSV disease in adults can help guide this clinical decision-making.

Disease severity from an infection can be affected by host immunity, pathogen virulence, as well as use of therapeutics targeting either the host response or the pathogen.^8^ Vaccination strengthens host immunity against infection and its sequelae and has been shown to attenuate both COVID-19 and influenza disease severity.^9,10,11^ Because vaccines against COVID-19 and influenza are routinely used by adults in the US, a comparison of disease severity caused by RSV with that of COVID-19 or influenza, by vaccination status, could be useful for framing the potential benefits of RSV vaccination, which may include reduction in disease severity, as observed with COVID-19 and influenza vaccination.

We assessed disease severity among adults hospitalized in the US with RSV during the 16 months immediately preceding recommendations by the Advisory Committee on Immunization Practices for RSV vaccine use. To provide context for the observed severity of RSV disease in hospitalized patients, we compared it with the severity of adults hospitalized with COVID-19 and influenza disease, stratified by vaccination status, during the same period.

## Methods

This cohort study was determined to be public health surveillance, with a waiver of ethics board review and participant informed consent by each participating institution and the CDC and was conducted consistent with applicable federal law and CDC policy (45 CFR part 46.102(l)(2), 21 CFR part 56; 42 USC §241(d); 5 USC §552a; 44 USC §3501, et seq). This study is reported following the Strengthening the Reporting of Observational Studies in Epidemiology (STROBE) reporting guideline.

### Design and Setting

We prospectively enrolled patients admitted to any of 25 hospitals in 20 US states within the Investigating Respiratory Viruses in the Acutely Ill (IVY) Network. The IVY Network has been continuously conducting observational analyses on respiratory viruses since 2019, is funded by CDC, is coordinated by Vanderbilt University Medical Center, and includes enrolling centers geographically dispersed throughout the continental US (eAppendix 1 in Supplement 1).^10,11,12,13^ Enrollment of patients with influenza and COVID-19 began in 2019 and 2020, respectively. Enrollment of patients with RSV began on February 1, 2022. The current analysis included patients enrolled between February 1, 2022, and May 31, 2023, which was the 16 months preceding recommendations for adult RSV vaccines in the US. This analysis expands on information previously published, which was limited to adults aged 60 years and older by assessing different clinical outcomes, stratified by vaccination status, as well as differences by virus subtype and phylogeny among all adults aged 18 years and older hospitalized within the IVY Network during same period.^14^

### Participants

Hospital admissions at each site were reviewed daily and assessed for eligibility. Hospitalized adults aged 18 years and older with symptoms or signs compatible with acute respiratory illness (ARI) who underwent clinical testing for RSV, SARS-COV-2, or influenza as standard of care were eligible for enrollment. ARI was defined as presence of at least 1 of the following: fever, cough, dyspnea, chest imaging findings consistent with pneumonia, or hypoxemia (ie, an oxygen saturation as measured by pulse oximetry <92% or supplemental oxygen use for patients without chronic oxygen needs, or escalation of oxygen therapy for patients who receive chronic supplemental oxygen). Nasal specimens were also obtained from enrolled patients and tested at a central laboratory (Vanderbilt University Medical Center) by reverse transcription–polymerase chain reaction (RT-PCR) for RSV, SARS-CoV-2, and influenza. Patients with nasal specimens with positive test results for RSV, SARS-CoV-2 or influenza within 10 days of illness onset and 3 days of hospital admission, based on either clinical or central laboratory testing, were included.

### Data Collection

Medical records were reviewed by trained personnel who abstracted demographic and clinical data, including in-hospital outcomes within 28 days of admission. Patients (or proxies) were interviewed for information on race, ethnicity, current illness, COVID-19 and influenza vaccination status, residence in a long-term care facility, and health care utilization in the previous year. Race and ethnicity were categorized as Black or African American, non-Hispanic; Hispanic or Latino, any race; White, non-Hispanic; other race, non-Hispanic (includes Asian, Native American or Alaska Native, and Native Hawaiian or Other Pacific Islander); and other (includes patients who self-reported their race and ethnicity as other and those for whom race and ethnicity were unknown). Data on race and ethnicity were collected because the association between respiratory viral infection and severe outcomes may vary by race or ethnicity.

The enrollment period for this analysis was prior to the availability of RSV vaccines; hence all patients with RSV infection were unvaccinated. Vaccination status for COVID-19 and influenza were obtained from electronic medical records (EMRs), state or jurisdictional registries, and by self-report. Final vaccination status was determined by combining data from verified documented sources (EMR and registry data) as well as plausible self-report based on date of vaccination.^13^ For this analysis, enrolled patients were excluded if they had confirmed or inconclusive laboratory test results indicating coinfection or possible coinfection with RSV, SARS-CoV-2, or influenza; had unknown vaccination status for COVID-19 or influenza when hospitalized with the respective virus; had only 1 mRNA vaccine (BNT162b2 [Pfizer-BioNTech] or mRNA-1273 [Moderna]) dose or 1 recombinant S protein vaccine (NVX-CoV2373 [Novavax]) dose when hospitalized with COVID-19; had unknown in-hospital outcomes; or were identified as ineligible for enrollment after data cleaning.

### Classification of Vaccination Status

Patients were classified into 2 COVID-19 vaccination groups: unvaccinated, defined as no prior receipt of COVID-19 vaccination; and vaccinated, if they had received at least a primary COVID-19 vaccine series or bivalent COVID-19 vaccine dose at least 7 days before illness onset (eMethods in Supplement 1). Patients who received bivalent COVID-19 vaccination may have previously received 1 to 5 doses of the original (ancestral strain) monovalent vaccines. Influenza vaccination status was classified into 2 groups: unvaccinated, defined as no receipt of influenza vaccine during the current season based on admission date or vaccinated, if they had received the current season’s influenza vaccination at least 14 days before illness onset.

### In-Hospital Outcomes Within 28 Days of Admission

Disease severity was characterized using clinical data during the patient’s hospital admission, beginning at initial hospital presentation, and ending at the earliest of hospital discharge, patient death, or the end of hospital day 28. Using these data, we created 6 nonmutually exclusive in-hospital outcomes: (1) supplemental oxygen therapy, defined as supplemental oxygen at any flow rate and by any device for patients not receiving chronic oxygen therapy or with escalation of oxygen therapy for patients receiving chronic oxygen therapy; (2) respiratory failure treated with advanced respiratory support, defined as a composite of new receipt of high-flow nasal cannula (HFNC), noninvasive ventilation (NIV) or invasive mechanical ventilation (IMV); (3) acute organ failure, defined as a composite of respiratory failure (new receipt of HFNC, NIV, or IMV), cardiovascular failure (use of vasopressors), or kidney failure (new receipt of kidney replacement therapy); (4) intensive care unit (ICU) admission; (5) hospital-free days to day 28, which is an ordinal composite of in-hospital death and hospital length of stay, defined as the number of days alive and out of the hospital between admission and 28 days later, with in-hospital death coded as −1^15^; and (6) a composite of IMV or death (eMethods in Supplement 1).

In addition to these nonmutually exclusive outcomes, we also generated mutually exclusive outcome categories based on a hierarchy of respiratory disease severity. An ordinal outcome, peak respiratory disease severity, was constructed with the following 5 categories: no oxygen therapy, standard-flow oxygen therapy, HFNC or NIV, IMV, and death. Each patient was classified into 1 category based on the highest category achieved during the hospitalization through day 28.

### Laboratory Analysis

Nasal swabs were obtained from enrolled patients and tested for RSV, SARS-CoV-2, and influenza by RT-PCR at a central laboratory (Vanderbilt University Medical Center, Nashville, Tennessee) (eMethods in Supplement 1). Viral whole genome sequencing was performed at the University of Michigan on specimens with positive results by central RT-PCR testing for RSV, SARS-CoV-2, or influenza (eMethods in Supplement 1 and eAppendix 2 in Supplement 2). Maximum likelihood phylogenetic trees were generated using IQ-TREE with a GTR model and visualized and annotated using ggtree.^16^ Strain names and Global Initiative on Sharing All Influenza Data accession identifications are provided in eAppendix 3 Supplement 3.

### Statistical Analysis

Demographics, clinical characteristics, and outcomes of enrolled patients were described by infecting virus (RSV, SARS-CoV-2, influenza) as well as by viral subtype (A and B subtypes for RSV; Omicron sublineages BA.1, BA.2, BA.4/5, BQ.1, and XBB.1.5 for SARS-CoV-2; and influenza A[H3N2] and A[H1N1]) and vaccination status (unvaccinated or vaccinated against COVID-19 or influenza). In-hospital outcomes were compared between patients hospitalized with RSV, COVID-19, and influenza disease among enrolled patients. A series of multivariable regression models were used to compare outcomes of patients hospitalized with RSV disease with 4 comparator groups: (1) patients hospitalized with COVID-19 without prior COVID-19 vaccination (unvaccinated COVID-19 group), (2) patients hospitalized with COVID-19 and previous COVID-19 vaccination (vaccinated COVID-19 group), (3) patients hospitalized with influenza disease without prior vaccination with the current season’s influenza vaccine (unvaccinated influenza group), and (4) patients hospitalized with influenza with receipt of the current season’s influenza vaccine (vaccinated influenza group). Models were adjusted for age, sex, self-reported race and ethnicity, number of organ systems with a chronic medical condition (eTable 1 in Supplement 1), and geographic region (US Department of Health and Human Services Region). Dichotomous outcomes were analyzed with multivariable logistic regression models. Ordinal outcomes, including hospital-free days and peak respiratory disease severity, were analyzed with multivariable proportional odds models. Because of the potential for type I error owing to multiple comparisons, findings from secondary analyses should be interpreted as exploratory. Statistical significance was indicated by a 2-sided *P* < .05.

In a sensitivity analysis, we repeated the severity comparisons for RSV, COVID-19, and influenza while restricting the population to adults aged 60 years and older. Currently, RSV vaccines are only recommended for adults aged 60 years and older, although adults of different ages may be considered in the future. Thus, we reported results for adults of all ages (≥18 years) as the primary analysis and results for adults aged 60 years and older as a secondary analysis.

Only patients with complete data for models were analyzed; missing data were not imputed. The numbers of patients with missing data were reported. All analyses were conducted using SAS software version 9.4 (SAS Institute). Data were analyzed from August to October 2023.

## Results

Between February 1, 2022 and May 31, 2023, a total of 9117 adults aged 18 years and older hospitalized with ARI had laboratory-confirmed RSV, SARS-CoV-2, or influenza based on either clinical or central laboratory testing and were enrolled. This included 34 patients (0.5%) identified with RSV after central testing of 6759 enrolled patients who did not undergo clinical testing for RSV (eFigure 1 in Supplement 1). Among 9117 patients with clinical or central laboratory-confirmed RSV, SARS-CoV-2, or influenza infection, 1119 were excluded from this analysis primarily due to confirmed coinfection (200 patients) (eTable 2 in Supplement 1), possible coinfection (376 patients), patients with COVID-19 with only 1 mRNA or 1 recombinant S protein vaccine dose (230 patients), unknown COVID-19 or influenza vaccination history (198 patients), or unknown clinical outcomes (70 patients) (eFigure 2 in Supplement 1). The final sample included 7998 adults (median [IQR] age, 67 [54-78] years; 4047 [50.6%] female).

### Participants

Among 7998 included patients with test results positive for RSV, COVID-19, or influenza, 484 (6.1%) were hospitalized with RSV, 6422 (80.3%) were hospitalized with COVID-19, and 1092 (13.7%) were hospitalized with influenza. Peak hospitalizations for both RSV and influenza occurred during November to December 2022, whereas high numbers of COVID-19 hospitalizations occurred throughout the analysis period (eFigure 3 and eFigure 4 in Supplement 1).

Adults hospitalized with RSV were younger than those hospitalized with COVID-19 (median [IQR] age, 65 [53-75] years vs 68 [56-78] years; *P* = .002), with no difference compared with those hospitalized with influenza disease (median [IQR] age, 64 [50-74] years; *P* = .09) (Table 1). A higher percentage of patients hospitalized with RSV had an ethnicity and race described as non-Hispanic Black compared with those hospitalized with COVID-19 (23.8% vs 19.4%; *P* < .01), but this percentage was similar among influenza patients (115 patients [23.8%] vs 271 patients [27.9%]; *P* = .34). Patients hospitalized with RSV and COVID-19 had similar proportions of underlying immunocompromising conditions (99 patients [20.5%] vs 1135 patients [17.7%]; *P* = .12), but patients with RSV were more likely to have immunocompromising conditions than influenza patients (149 patients [13.6%]; *P* < .001). Chronic cardiovascular and pulmonary conditions were common among patients with each of the viruses. Patients hospitalized with RSV were more likely to self-report dyspnea than patients with either COVID-19 (385 patients [79.6%] vs 3916 patients [61.0%]; *P* < .001) or influenza (788 patients [72.2%]; *P* = .002). Of 6422 patients with COVID-19, 5000 (77.9%) were classified as vaccinated and were a median (IQR) of 259 days (152-407) days from last COVID-19 vaccination. Of 1092 patients with influenza, 393 (36.0%) had received seasonal influenza vaccination.

**Table 1.** Characteristics of Adults Hospitalized With RSV, COVID-19, or Influenza

Characteristic	Patients, No. (%)		
	RSV (n = 484)	COVID-19 (n = 6422)	Influenza (n = 1092)
Age, y			
Median (IQR)	65 (53-75)	68 (56-78)	64 (50-74)
18-49	101 (20.9)	1145 (17.8)	271 (24.8)
50-59	84 (17.4)	883 (13.8)	157 (14.4)
60-69	114 (23.6)	1475 (23.0)	282 (25.8)
70-79	107 (22.1)	1500 (23.4)	217 (19.9)
≥80	78 (16.1)	1419 (22.1)	165 (15.1)
Race and ethnicity			
Black or African American, non-Hispanic	115 (23.8)	1245 (19.4)	305 (27.9)
Hispanic or Latino, any race	78 (16.1)	789 (12.3)	182 (16.7)
White, non-Hispanic	248 (51.2)	3932 (61.2)	517 (47.3)
Other race, non-Hispanic^a^	38 (7.9)	345 (5.4)	72 (6.6)
Other^b^	5 (1.0)	111 (1.7)	16 (1.5)
Sex			
Female	267 (55.2)	3183 (49.6)	597 (54.7)
Male	217 (44.8)	3239 (50.4)	495 (45.3)
HHS region^c^			
1	58 (12.0)	1175 (18.3)	146 (13.4)
2	42 (8.7)	285 (4.4)	101 (9.3)
3	14 (2.9)	329 (5.1)	15 (1.4)
4	98 (20.3)	1050 (16.4)	190 (17.4)
5	64 (13.2)	1096 (17.1)	218 (20.0)
6	37 (7.6)	672 (10.5)	141 (12.9)
7	49 (10.1)	389 (6.1)	75 (6.9)
8	75 (15.5)	754 (11.7)	147 (13.5)
9	29 (6.0)	385 (6.0)	28 (2.6)
10	18 (3.7)	287 (4.5)	31 (2.8)
Organ systems with a chronic medical condition^d^			
Median (IQR), No.	2 (2-3)	2 (1-3)	2 (1-3)
Cardiovascular disease	345 (71.3)	4603 (71.7)	741 (67.9)
Pulmonary disease	222 (45.9)	2019 (31.4)	454 (41.6)
Neurologic disease	60 (12.4)	1158 (18.0)	138 (12.6)
Gastrointestinal disease	25 (5.2)	507 (7.9)	71 (6.5)
Endocrine disease	184 (38.0)	2730 (42.5)	421 (38.6)
Kidney disease	125 (25.8)	1601 (24.9)	203 (18.6)
Hematologic disease	72 (14.9)	1024 (16.0)	136 (12.5)
Autoimmune disease	36 (7.4)	420 (6.5)	69 (6.3)
Immunocompromising conditions^e^	99 (20.5)	1135 (17.7)	149 (13.6)
Resident of long-term care facility	40 (8.3)	488 (7.6)	60 (5.5)
Outpatient medical visits in previous, median (IQR), No.	4 (3-5)	4 (3-5)	3 (2-5)
Time from illness onset to hospital admission, median (IQR), d	3 (1-4)	2 (0-4)	2 (1-4)
Symptoms and signs			
Fever	168 (34.7)	2819 (43.9)	588 (53.9)
Cough	382 (78.9)	4138 (64.4)	855 (78.3)
Dyspnea	385 (79.6)	3916 (61.0)	788 (72.2)
Chest imaging with evidence of pneumonia	132 (27.3)	1423 (22.2)	250 (22.9)
COVID-19 vaccination status^f^			
Unvaccinated	78 (16.1)	1422 (22.1)	281 (25.7)
Vaccinated^g^	402 (83.0)	5000 (77.9)	798 (73.1)
Influenza vaccination status^h^			
Unvaccinated	226 (46.7)	3060 (47.7)	699 (64.0)
Vaccinated^i^	216 (44.6)	2882 (44.9)	393 (36.0)

Abbreviations: HHS, US Department of Health and Human Services; RSV, respiratory syncytial virus.

^a^ Other race, non-Hispanic includes Asian, Native American or Alaska Native, and Native Hawaiian or Other Pacific Islander, which were combined because of small counts.

^b^ Other includes patients who self-reported their race and ethnicity as other and those for whom race and ethnicity were unknown.

^c^ Hospitals by US Department of HHS Region included region 1: Baystate Medical Center (Springfield, Massachusetts), Beth Israel Deaconess Medical Center (Boston, Massachusetts); Yale University (New Haven, Connecticut); region 2: Montefiore Medical Center (New York City borough of the Bronx, New York); region 3: Johns Hopkins Hospital (Baltimore, Maryland); region 4: Emory University Medical Center (Atlanta, Georgia), University of Miami Medical Center (Miami, Florida), Vanderbilt University Medical Center (Nashville, Tennessee), Wake Forest University Baptist Medical Center (Winston-Salem, North Carolina); region 5: Cleveland Clinic (Cleveland, Ohio), Hennepin County Medical Center (Minneapolis, Minnesota), Henry Ford Health (Detroit, MI); The Ohio State University Wexner Medical Center (Columbus, Ohio), University of Michigan Hospital (Ann Arbor, Michigan); region 6: Baylor Scott & White Medical Center (Temple, Texas), Baylor University Medical Center (Dallas, Texas); region 7: Barnes-Jewish Hospital (St. Louis, Missouri), University of Iowa Hospitals (Iowa City, Iowa); region 8: Intermountain Medical Center (Murray, Utah) and UCHealth, University of Colorado Hospital (Aurora, Colorado); region 9: University of Arizona Medical Center (Phoenix, Arizona), Stanford University Medical Center (Stanford, California), UCLA Medical Center (Los Angeles, California); and region 10: Oregon Health & Science University Hospital (Portland, Oregon), University of Washington (Seattle, Washington).

^d^ Details of conditions within each group are listed in eTable 1 in Supplement 1.

^e^ Immunocompromising conditions were defined as active solid tumor or hematologic malignancy (ie, newly diagnosed malignancy or treatment for a malignancy within the previous 6 months), solid organ transplant; bone marrow or hematopoietic stem cell transplant, HIV infection, congenital immunodeficiency syndrome; or use of an immunosuppressive medication within the previous 30 days.

^f^ A total of 17 patients (0.2%) were missing COVID-19 vaccination status, including 4 patients with RSV (0.8%), 0 patients with COVID-19, and 13 patients with influenza (1.2%).

^g^ Includes patients with receipt of the original (ancestral strain) monovalent vaccines, specifically at least 2 doses of BNT1262b2, (Pfizer-BioNTech), mRNA-1273 (Moderna), or NVX-CoV2373 (Novavax), or at least 1 dose of Ad26.COV2.S (Janssen) or at least 1 dose of BNT1262b2 bivalent vaccine and mRNA-1273.222 bivalent vaccine. Patients who received bivalent vaccination may have previously received 1 to 5 doses of the original (ancestral strain) monovalent vaccines. The median (IQR) days from last COVID-19 vaccination to admission date among COVID-19 patients is 259 (152-407) days.

^h^ A total of 522 patients (6.5%) were missing influenza vaccination status, including 42 patients with RSV (8.7%), 480 patients with COVID-19 (7.5%), and 0 patients with influenza.

^i^ Patients were classified as vaccinated against influenza if they received the current season’s influenza vaccination based on the period in which they were enrolled.

### Discrete Severity Outcomes

Overall, most outcomes revealed disease severity among patients with RSV that was not significantly different from patients with unvaccinated COVID-19 and influenza and substantially higher than patients with vaccinated COVID-19 and influenza (Table 2). For example, the outcome of IMV or death was experienced by 58 of 484 patients with RSV (12.0%); 201 of 1422 unvaccinated patients with COVID-19 (14.1%) (adjusted odds ratio [aOR], 0.82; 95% CI, 0.59-1.13); 458 of 5000 vaccinated patients with COVID-19 (9.2%) (aOR, 1.38; 95% CI, 1.02-1.86); 72 of 699 unvaccinated patients with influenza (10.3%) (aOR, 1.20; 95% CI, 0.82-1.76); and 20 of 393 vaccinated patients with influenza (5.1%) (aOR, 2.81; 95% CI, 1.62-4.86). Patients with RSV were more than twice as likely to receive advanced respiratory support than vaccinated patients with COVID-19 (aOR, 2.03; 95% CI, 1.64-2.51) or vaccinated patients with influenza (aOR, 2.71; 95% CI, 1.89-3.87). Frequencies and proportions of each outcome and their components are shown in eTable 3 in Supplement 1.

**Table 2.** Severity of RSV-Associated Hospitalizations vs COVID-19– or Influenza-Associated Hospitalizations, by Vaccination Status, Among US Adults

In-hospital outcomes	Patients, No. (%)				RSV vs COVID-19 by vaccination status				Patients with influenza, No. (%)		RSV vs influenza by vaccination status				
	RSV (n = 484)	COVID-19													
		Unvaccinated (n = 1422)	Vaccinated (n = 5000)^a^	Unvaccinated			Vaccinated^a^		Unvaccinated (n = 699)	Vaccinated (n = 393)^b^	Unvaccinated		Vaccinated^b^	
				aOR (95% CI)^c^		*P* value	aOR (95% CI)^c^	*P* value			aOR (95% CI)^c^	*P* value	aOR (95% CI)^c^	*P* value
Supplemental oxygen therapy^d^	355 (73.4)	857 (60.3)	2924 (58.5)	1.82 (1.42-2.32)		<.001	2.16 (1.74-2.68)	<.001	460 (65.8)	249 (63.4)	1.27 (0.97-1.68)	.09	1.86 (1.36-2.55)	<.001
Advanced respiratory support^e^	146 (30.2)	332 (23.4)	888 (17.8)	1.40 (1.10-1.78)		.006	2.03 (1.64-2.51)	<.001	157 (22.5)	57 (14.5)	1.47 (1.12-1.93)	.006	2.71 (1.89-3.87)	<.001
Acute organ failure^f^	152 (31.4)	359 (25.3)	1015 (20.3)	1.32 (1.05-1.68)		.02	1.84 (1.49-2.26)	<.001	170 (24.3)	61 (15.5)	1.38 (1.05-1.81)	.02	2.62 (1.85-3.71)	<.001
ICU admission	120 (24.8)	326 (22.9)	847 (16.9)	1.11 (0.86-1.43)		.43	1.55 (1.24-1.95)	<.001	152 (21.8)	42 (10.7)	1.14 (0.86-1.53)	.36	2.65 (1.78-3.95)	<.001
Hospital-free days, median (IQR)^g^	23 (18-25)	23 (17-25)	23 (19-25)	1.18 (0.98-1.42)^h^		.08	0.85 (0.72-1.00)^h^	.05	24 (20-26)	24 (22-26)	0.79 (0.64-0.97)^h^	.03	0.53 (0.42-0.68)^h^	<.001
IMV or death	58 (12.0)	201 (14.1)	458 (9.2)	0.82 (0.59-1.13)		.22	1.38 (1.02-1.86)	.03	72 (10.3)	20 (5.1)	1.20 (0.82-1.76)	.35	2.81 (1.62-4.86)	<.001

Abbreviations: aOR, adjusted odds ratio; ICU, intensive care unit; IMV, invasive mechanical ventilation; RSV, respiratory syncytial virus.

^a^ Includes patients with receipt of the original (ancestral strain) monovalent vaccines, specifically at least 2 doses of BNT1262b2, (Pfizer-BioNTech), mRNA-1273 (Moderna), or NVX-CoV2373 (Novavax), or at least 1 dose of Ad26.COV2.S (Janssen) or at least 1 dose of BNT1262b2 Bivalent vaccine and mRNA-1273.222 bivalent vaccine. Patients who received bivalent vaccination may have previously received 1 to 5 doses of the original (ancestral strain) monovalent vaccines.

^b^ Patients were classified as vaccinated against influenza if they received the current season’s influenza vaccine based on the period in which they were enrolled.

^c^ Multivariable logistic regression models were adjusted for age, sex, race and ethnicity, number of organ systems with chronic medical conditions and US Department of Health & Human Services region.

^d^ Supplemental oxygen therapy was defined as use of supplemental oxygen at any flow rate with any device for those not on chronic supplemental oxygen, or as escalation of respiratory support for patients who use chronic supplemental oxygen, at any time during hospitalization prior to day 28.

^e^ Advanced respiratory support was defined as receipt of organ support for respiratory failure (ie, acute use of HFNC, noninvasive ventilation, or IMV) at any time during the hospitalization before day 28. Patients receiving chronic home noninvasive ventilation were classified as requiring respiratory support if they received IMV in the hospital. Patients receiving chronic home IMV were ineligible for this outcome.

^f^ Acute organ failure is a composite of respiratory failure (ie, acute use of high-flow nasal cannula, noninvasive ventilation, and IMV), cardiovascular failure (ie, use of vasopressors) or renal failure (ie, acute use of kidney replacement therapy).

^g^ Hospital-free days to day 28 is a composite of in-hospital death and hospital length of stay defined as the number of days alive and out of the hospital between admission and 28 days later. Patients who died during the hospitalization are classified as having −1 hospital-free days and those who were hospitalized for more than 28 days were classified as having zero hospital-free days. For patients discharged alive before day 28, hospital-free days were calculated as 28 minus the length of stay to generate an ordinal scale.

^h^ Because hospital-free days is an ordinal outcome, multivariable proportional odds models were used to estimate the association of hospital-free days between RSV and unvaccinated or vaccinated COVID-19 or influenza. Models were adjusted for the same covariables used in multivariable logistic regression models, including age, sex, race and ethnicity, number of organ systems with chronic medical conditions and US Department of Health & Human Services region.

Similar results were found when comparing RSV disease severity with COVID-19 and influenza disease among adults aged 60 years and older (eTable 4 and eTable 5 in Supplement 1).

### Peak Respiratory Severity Ordinal Outcome

**Figure 1.**
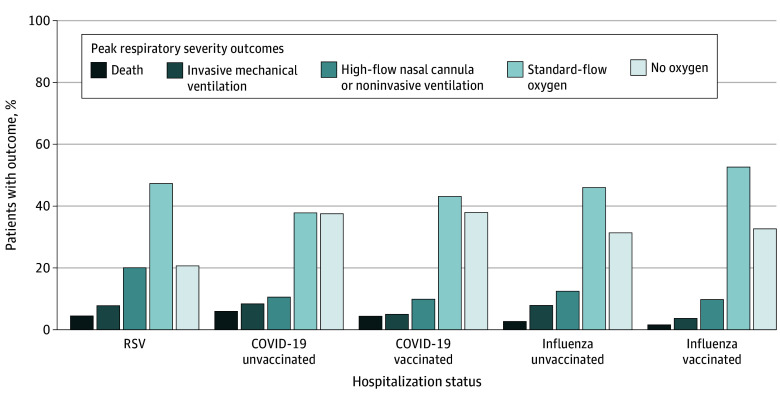
Peak Respiratory Severity of Adults Hospitalized With Respiratory Syncytial Virus (RSV), COVID-19, or Influenza by Vaccination Status Peak respiratory severity was classified by an ordinal scale as follows: (1) no oxygen therapy; (2) standard-flow oxygen therapy (<30 L/min); (3) high-flow nasal cannula (≥30 L/min) or noninvasive ventilation; (4) invasive mechanical ventilation; and (5) death.

When evaluating peak respiratory disease severity, patients with RSV had higher overall severity compared with the unvaccinated COVID-19 group (aOR, 1.54; 95% CI, 1.27-1.86) and the unvaccinated influenza group (aOR, 1.48; 95% CI, 1.18-1.85) and substantially higher overall disease severity compared with the vaccinated COVID-19 group (aOR, 2.16; 95% CI, 1.82-2.57) and the vaccinated influenza group (aOR, 2.40; 95% CI, 1.83-3.14) (Figure 1).

### RSV Subtypes, SARS-CoV-2 Lineages, and Influenza Subtypes

**Figure 2.**
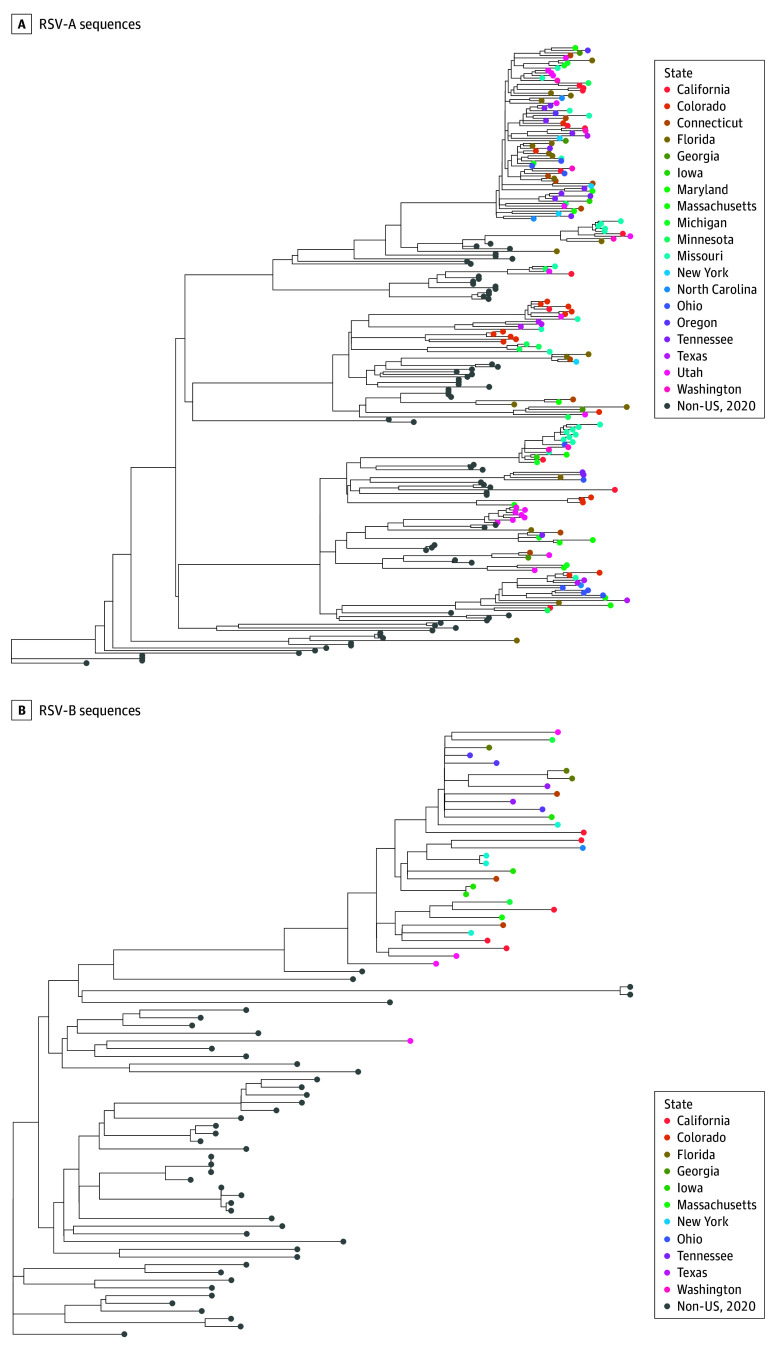
Maximum Likelihood Phylogenetic Trees A, 177 RSV-A sequences from adults aged 18 years and older hospitalized within the Investigating Respiratory Viruses in the Acutely Ill Network of 25 hospitals in 20 US States (tips color coded by State) and 68 contextual sequences from outside the United States collected between January and March 2020, available on Global Initiative on Sharing All Influenza Data. B, 32 RSV-B sequences from adults aged 18 years and older hospitalized within the Investigating Respiratory Viruses in the Acutely Ill Network of 25 hospitals in 20 US States (tips color coded by State) with 46 contextual sequences from outside the United States collected between January and March 2020, available on Global Initiative on Sharing All Influenza Data.

Of 368 patients with RSV subtype identified, 250 (67.9%) were subtype A and 118 (32.1%) were subtype B. Whole-genome sequencing of RSV subtypes A and B found that lineages circulating during the period of this analysis derived from lineages circulating in early 2020 (before the COVID-19 pandemic in the US) (Figure 2). Clinical outcomes were overall similar between RSV subtype A and B (Table 3). All patients hospitalized with COVID-19 had SARS-CoV-2 Omicron lineages. Among 3466 patients with identified SARS-CoV-2 lineages, BA.1 was the least frequent Omicron lineage (217 patients [6.3%]) but demonstrated the greatest severity (Table 3). Among 649 patients hospitalized with influenza infection and identified subtype, influenza A(H3N2) predominated during the analysis period (474 patients [73.0%]) and had lower or similar severity than influenza A(H1N1) (Table 3).

**Table 3.** Disease Severity of RSV, SARS-CoV-2, and Influenza by Subtype or Lineage Among US Adults

In-hospital outcome	RSV subtypes			SARS-CoV-2 Omicron lineages						Influenza A Subtypes		
	Patients, No. (%)		*P* value	Patients, No. (%)					*P* value	Patients, No. (%)		*P* value
	A (n = 250)	B (n = 118)		BA.1 (n = 217)	BA.2 (n = 691)	BA.4/5 (n = 1310)	BQ.1 (n = 467)	XBB.1.5 (n = 781)		A(H3N2) (n = 474)	A(H1N1) (n = 175)	
Supplemental oxygen therapy^a^	188 (75.2)	84 (71.2)	.41	158 (72.8)	372 (53.8)	751 (57.3)	275 (58.9)	443 (56.7)	<.001	297 (62.7)	121 (69.1)	.13
Advanced respiratory support^b^	71 (28.4)	32 (27.1)	.80	70 (32.3)	87 (12.6)	238 (18.2)	77 (16.5)	136 (17.4)	<.001	76 (16.0)	42 (24.0)	.02
Acute organ failure^c^	74 (29.6)	32 (27.1)	.62	76 (35.0)	102 (14.8)	258 (19.7)	87 (18.6)	159 (20.4)	<.001	84 (17.7)	44 (25.1)	.04
ICU admission	62 (24.8)	22 (18.6)	.19	59 (27.2)	93 (13.5)	213 (16.3)	75 (16.1)	108 (13.8)	<.001	68 (14.4)	32 (18.3)	.22
IMV or death	28 (11.2)	11 (9.3)	.58	34 (15.7)	49 (7.1)	126 (9.6)	40 (8.6)	60 (7.7)	.002	33 (7.0)	14 (8.0)	.65

Abbreviations: ICU, intensive care unit; IMV, invasive mechanical ventilation; RSV, respiratory syncytial virus.

^a^ Supplemental oxygen therapy was defined as use of supplemental oxygen at any flow rate with any device for those not on chronic supplemental oxygen, or as escalation of respiratory support for patients who use chronic supplemental oxygen, at any time during hospitalization prior to day 28.

^b^ Advanced respiratory support was defined as receipt of organ support for respiratory failure (high-flow nasal cannula, noninvasive ventilation, or IMV) at any time during the hospitalization before day 28. Patients receiving chronic home noninvasive ventilation were classified as requiring respiratory support if they received IMV in the hospital. Patients receiving chronic home IMV were ineligible for this outcome.

^c^ Acute organ failure is a composite of respiratory failure (including use of high-flow nasal cannula, noninvasive ventilation, and IMV), cardiovascular failure (use of vasopressors), or renal failure (acute use of kidney replacement therapy).

## Discussion

In this prospective, multicenter cohort study of adults hospitalized in 20 US states during the 16 months preceding the firstUS adult RSV vaccination recommendation, the frequency of RSV hospitalizations was substantially lower than for COVID-19 and influenza; RSV hospitalizations were approximately one-fourteenth as common as COVID-19 hospitalizations and one-half as common as influenza hospitalization. Disease severity of RSV was similar to COVID-19 and influenza among unvaccinated patients and higher than COVID-19 and influenza among vaccinated patients hospitalized with those diseases for the critical outcomes of ICU admission and IMV or death. RSV genomes sequenced from this cohort derived from early 2020 lineages, suggesting the virus circulating during the period of this analysis was similar to RSV that circulated before the COVID-19 pandemic. This evaluation of RSV epidemiology during a period of endemic COVID-19 demonstrates that RSV is a serious respiratory infection in adults, and especially older adults.^14^ Newly approved RSV vaccines for adults aged 60 years and older have the potential to reduce this severity, similar to attenuation of disease severity achieved with COVID-19 and influenza vaccination, as previously reported^9,10,11^ and also observed in this analysis.

Although several studies have previously compared severity of RSV with influenza disease, strengths of this analysis include comparisons of RSV (which was not vaccine preventable during the period of this analysis) with unvaccinated COVID-19 and influenza disease to separate the association of vaccination in attenuating disease severity from the direct effects of the pathogen. By stratifying our COVID-19 and influenza populations by vaccination status, we show that critical outcomes of ICU admission and IMV or death occurred in a similar proportion of unvaccinated adults hospitalized with RSV compared with unvaccinated adults hospitalized with COVID-19 or influenza. Although outcome definitions varied across studies and analyses were not stratified by vaccination status in prior studies, most prior work suggested RSV disease was more severe than influenza disease among hospitalized adults, including use of supplemental oxygen, IMV, or ICU admission.^17,18,19,20^

Three prior studies have compared RSV disease severity with COVID-19 and suggested lower severity for RSV compared with COVID-19.^19,21,22^ However, 2 of these studies^19,21^ were conducted using data from 2020, when COVID-19 vaccines were not available and Omicron lineages were not in circulation. Between 2020 and 2022 to 2023, the clinical manifestations of COVID-19 evolved considerably due to the emergence of the Omicron variant, increases in population-level immunity from both vaccination and infection, and improvements in clinical care, including increases in use of antiviral treatments.^23,24^ As a consequence, the severity of COVID-19 has declined, and the severity of RSV disease is now relatively high compared with COVID-19 from Omicron lineages.

An additional strength of this analysis is that respiratory specimens were obtained from participants, which allowed for subsequent characterization into subtypes by RT-PCR and into lineages based on whole genome sequencing. RSV A and B subtypes have been shown to cocirculate, although 1 subtype often predominates in each season.^25^ There are limited data comparing severity between RSV A and B subtypes in adults. Our findings demonstrate very similar patient characteristics and clinical outcomes between RSV A and B subtypes, consistent with studies from before the COVID-19 pandemic.^26,27^ Similar to prior work during the 2022 RSV surge, we demonstrated expected RSV genomic divergence from early 2020 lineages, suggesting that the severity of RSV disease observed in this analysis is unlikely to have resulted from major genomic changes in RSV during the COVID-19 pandemic.^28^

The high disease severity observed among older adults without previous RSV vaccination in this analysis is important to guide decision-making for RSV vaccination in this population. Both clinical trials that led to Food and Drug Administration approval of RSV vaccines for adults aged 60 years and older showed moderate to high efficacy of RSV vaccination against lower respiratory tract disease, which is in the causal pathway leading to severe disease.^29,30^ Although additional studies are needed to assess the protection of these vaccines against severe respiratory disease in older adults, our results suggest that there is a burden of disease beyond prevention of RSV hospitalization—the reduction of in-hospital RSV disease severity—that could occur with RSV vaccination, as shown for COVID-19 and influenza vaccination in both previous studies and this analysis.^9,10,11^

### Limitations

This analysis is subject to limitations. First, it is possible that RSV was preferentially detected among patients who were more severely ill and therefore more likely to receive clinical testing for RSV at participating hospitals and be subsequently enrolled. However, most enrolled patients had nasal swabs tested at a central laboratory for RSV, SARS-CoV-2, and influenza, lessening the potential bias of detecting RSV among patients who were more severely ill based on clinical testing only. During the period of this analysis, we enrolled 6759 adults aged 18 years and older hospitalized with ARI who did not have clinical testing for RSV; only 34 (0.5%) of these patients had a positive RSV test result based on central testing, suggesting the number of undetected RSV hospitalizations was likely low. Second, treatment with antiviral and immunomodulatory medications was not considered in analyses. Quantifying the effect of treatment on observed severity and accounting for it in severity comparisons is complicated by several factors, including that there are currently no routine treatments available RSV; indications for COVID-19 inpatient treatment is often based on severity, making it difficult to disentangle the associations between treatment and observed severity; and COVID-19 treatment practice varied considerably during the analysis period. We presented respiratory virus groups (RSV, COVID-19, and influenza) stratified by vaccination status without stratification or adjustment for acute treatments; the presented severity levels for COVID-19 and influenza represent a mix of patients who did and did not receive antiviral and immunomodulatory treatments.

## Conclusions

In this cohort study among adults hospitalized in the US during the 16 months preceding recommendations for the first adult RSV vaccines, RSV disease severity was similar to severity of COVID-19 and influenza disease among unvaccinated patients and substantially higher than COVID-19 and influenza disease among vaccinated patients. Severity of RSV disease among adults may be important to consider as RSV vaccine policy evolves.

## Notes

Supplement 1. **eAppendix 1.** Investigators and Collaborators
eMethods.
**eTable 1.** Underlying Medical Conditions Obtained Through Medical Record Review **eTable 2.** Characteristics and In-Hospital Outcomes of Adults Aged ≥18 Years Hospitalized With Coinfections of RSV, SARS-CoV-2, or Influenza **eTable 3.** Components of Composite in-Hospital Outcomes Among Adults Aged ≥18 Years Hospitalized With RSV, COVID-19, or Influenza by Vaccination Status **eTable 4.** Severity of RSV-Associated Hospitalizations vs COVID-19-Associated Hospitalizations, by Vaccination Status, Among US Adults Aged ≥60 Years **eTable 5.** Severity of RSV-Associated Hospitalizations vs Influenza-Associated Hospitalizations, by Vaccination Status, Among US Adults Aged ≥60 Years **eFigure 1.** RSV Testing Among Adults Aged ≥18 Years Hospitalized With Acute Respiratory Illness (ARI) **eFigure 2.** Adults Aged ≥18 Years Hospitalized With RSV, COVID-19, or Influenza **eFigure 3.** Frequency of Adults Aged ≥18 Years Hospitalized With RSV, COVID-19, or Influenza Disease by Admission Week **eFigure 4.** Frequency of Adults Aged ≥18 Years Hospitalized With RSV, COVID-19, or Influenza Disease by Admission Week and US Department of Health and Human Services (HHS) Region

Supplement 2. **eAppendix 2.** RSV Primer Pools

Supplement 3. **eAppendix 3.** RSV Strain Names and GISAID Accession IDs

Supplement 4.
Data Sharing Statement
