# Supplementary material for: Severity of Respiratory Syncytial Virus vs COVID-19 and Influenza Among Hospitalized US Adults
Source: JAMA Netw Open. 2024 Apr 4;7(4):e244954. doi: 10.1001/jamanetworkopen.2024.4954 (PMC11192181; doi:10.1001/jamanetworkopen.2024.4954)

Supplemental Online Content

Surie D, Yuengling K, DeCuir J, et al; Investigating Respiratory Viruses in the Acutely Ill (IVY) Network. Severity of respiratory syncytial virus vs COVID-19 and influenza among hospitalized US adults. *JAMA Netw Open*. 2024;7(4):e244954. doi:10.1001/jamanetworkopen.2024.4954

eAppendix 1. Investigators and Collaborators

eMethods.

eTable 1. Underlying Medical Conditions Obtained Through Medical Record Review

eTable 2. Characteristics and In-Hospital Outcomes of Adults Aged ≥18 Years Hospitalized With Coinfections of RSV, SARS-CoV-2, or Influenza

eTable 3. Components of Composite in-Hospital Outcomes Among Adults Aged ≥18 Years Hospitalized With RSV, COVID-19, or Influenza by Vaccination Status

eTable 4. Severity of RSV-Associated Hospitalizations vs COVID-19-Associated Hospitalizations, by Vaccination Status, Among US Adults Aged ≥60 years

eTable 5. Severity of RSV-Associated Hospitalizations vs Influenza-Associated Hospitalizations, by Vaccination Status, Among US Adults Aged ≥60 years

eFigure 1. RSV Testing Among Adults Aged ≥18 Years Hospitalized With Acute Respiratory Illness (ARI)

eFigure 2. Adults Aged ≥18 Years Hospitalized with RSV, COVID-19, or Influenza

eFigure 3. Frequency of Adults Aged ≥18 Years Hospitalized With RSV, COVID-19, or Influenza Disease by Admission Week

eFigure 4. Frequency of Adults Aged ≥18 Years Hospitalized With RSV, COVID-19, or Influenza Disease by Admission Week and US Department of Health and Human Services (HHS) Region

This supplemental material has been provided by the authors to give readers additional information about their work.

© 2024 Surie D et al. *JAMA Network Open.*

eAppendix 1.

IVY

INVESTIGATING RESPIRATORY VIRUSES IN THE ACUTELY ILL

Investigators and collaborators in the **I**nvestigating Respiratory **V**iruses in the Acutel**Y** Ill (IVY) Network

Baylor, Scott and White, Temple, Texas
Nicole Calhoun, Kempapura Murthy, Joselyn Cravens, Judy Herrick, Amanda McKillop, Eric Hoffman, Ashley Graves, Martha Zayed, Michael Smith, Tresa McNeal. Baylor Scott and White Health, Dallas: Cristie Columbus, Robert Gottleib, Catherine Raver, Tammy Fisher, Mariana Hurutado-Rodriguez, Taryn Kruse, Symone Dunkley, Gabriela Perez, Ashley Bychkowski, Therissa Grefsrud, Nicole Calhoun, Kempapura Murthy, Judy Herrick, Michael Smith

Baystate Medical Center, Springfield, Massachusetts
Jay Steingrub, Lesley De Souza, Scott Ouellette, Rae Lynn DeFeo, Denise Gallant

Beth Israel Medical Center, Boston Massachusetts
Nathan I. Shapiro, Ana Grafals, Carlo Ottanelli, Kimberly Redman, Alessio Barca, Madhavan Das, Jodens Didie, Shira Mann

Centers for Disease Control and Prevention (CDC), Atlanta, Georgia
Diya Surie, Jennifer DeCuir, Katharine A. Yuengling, Indrani Mukherjee, Meredith L. McMorrow, Nathaniel M. Lewis, Sascha Ellington

Cleveland Clinic, Cleveland, Ohio
Omar Mehkri, Megan Mitchell, Zachary Griffith, Connery Brennan, Kiran Ashok, Bryan Poynter, Abhijit Duggal

Emory University, Atlanta, Georgia
Laurence Busse, Caitlin ten Lohuis, Nicholas Stanley, Sophia Zhang

Hennepin County Medical Center, Minneapolis, Minnesota
Matthew E. Prekker, MD, Anne E.P. Frosch, MD, Heidi L. Erickson, Audrey Hendrickson, MPH, BS, Kowsar Hurreh, BS, Leyla A. Taghizadeh, Mary C. O'Rourke, Samuel Boes

Intermountain Medical Center, Murray, Utah
Ithan Peltan, Jenna Lumpkin, Cassie Smith, Shandi Poulsen, Jose Romero

Johns Hopkins University, Baltimore, Maryland

© 2024 Surie D et al. *JAMA Network Open.*

David N. Hager, Harith Ali, Minh, Phan

Montefiore Medical Center, Bronx, New York
Michelle Gong, Amira Mohamed, Rahul Nair, Jen-Ting (Tina) Chen

Ohio State Medical Center, Columbus, Ohio
Matthew Exline, Sarah Karow, Maryiam Khan, Madison So, Connor Snyder, Gabrielle Swoope, David Smith, Brooke Lee, Amanie Rasul, Manisha Pathak, Zachery Lewald, Reece Wilson

Oregon Health and Sciences University, Portland, Oregon
Akram Khan, José Peña, Genesis Briceno, Cassandra Ahmed, Jesus Martinez, Edvinas Pocius, Minn Oh, Jessica Hyde, Gopal Allada, Kinsley Hubel

Stanford University, Stanford, California
Jennifer G. Wilson, Cynthia Perez, Lily Lau, Grace Tam, Ismail Hakki Bekiroglu, Vanessa Pitre

University of Arizona
Jarrod Mosier, Beth Salvagio Campbell, Anitza Lopez, Mary Labus, Francis Nagore

University of California-Los Angeles, Los Angeles, California
Cody Tran, Omai Garner, Sukantha Chandrasekaran

University of Colorado, Aurora, Colorado
Adit Ginde, Amanda Martinez, Aimee Steinwand, Amy Sullivan, Cori Withers, Jacob Rademacher, Rachel Obradovich

University of Iowa, Iowa City, Iowa
Nicholas Mohr, Anne Zepeski, Paul Nassar, Noble Briggs, Jacob Hampton, Cathy Fairfield

University of Miami, Miami, Florida
Chris Mallow, Carolina Rivas

University of Michigan, Ann Arbor, Michigan
Emily Martin, Arnold Monto, Adam Lauring, Aleda Leis, EJ McSpadden, Rachel Truscon, Anne Kaniclides, Lara Thomas, Ramsay Bielak, Weronika Damek Valvano, Rebecca Fong, William J. Fitzsimmons, Christopher N. Blair, Julie Gilbert, Leigh Papalambros, Ankur Holz

University of Washington, Seattle, Washington
Nicholas Johnson, Sarah Stuckey, Vasisht Srinivasan, Christine D. Crider, Kyle A. Steinbock, Thomas C. Paulsen, Layla A. Anderson

Vanderbilt University Medical Center, Nashville, Tennessee
Wesley H. Self, H. Keipp Talbot, Carlos Grijalva, Ian Jones, Natasha Halasa, James Chappell, Kelsey Womack, Jillian Rhoads, Adrienne Baughman, Christy Kampe, Jakea Johnson, Sydney Swan, Cassandra Johnson, Yuwei Zhu, Todd Rice, Jonathan Casey, William B. Stubblefield, Yuwei Zhu, Laura L. Short, Lauren J. Ezzell, Margaret E. Whitsett, Rendie E. McHenry,

© 2024 Surie D et al. *JAMA Network Open.*

Samarian J. Hargrave, Marcia Blair, Jennifer L. Luther, Claudia Guevara Pulido, Bryan P. M. Peterson, Shanice L. Cummings, Emma Claire Gauthier

Wake Forest University, Winston-Salem, North Carolina
D. Clark Files, Kevin Gibbs, Leigha Landreth, Madeline Hicks, Lisa Parks, Fay Agyepong, Jacqueline Maycee Cain, Darija Ward

Washington University, St. Louis, Missouri
Jennie Kwon, Bijal Parikh, David McDonald, Carleigh Samuels, Lucy Vogt, Caroline O'Neil, Alyssa Valencia, Tiffany Hink, Olivia Arter, Kim Vu, Francesca Yerbic

Baylor, Scott, and White Baylor University Medical Center, Dallas, Texas
Ashley Bychkowski, BSN, RN, CIC, Symone Dunkley, BA, Tammy Fisher, MBA, MSN, RN, Therissa Grefsrud, MBA-HCM, BSN, RN, CIC, Mariana H. Hurutado-Rodriguez, RN, Gabriela Cantu Perez MPH, CIC

Henry Ford Health, Detroit, Michigan
Kim Beney, RHIT; Rachna Jayaprakash, MBBS, Sindhuja Koneru, PhD, MS; Jean Ashley Lava, MPH; Zina Pinderi, BS; Melissa Resk, MS

Yale University, New Haven, Connecticut
Anirudh Goyal, Lauren DeLamielleure, Michael Kosover

© 2024 Surie D et al. *JAMA Network Open.*

eMethods

1. Eligibility Criteria and Enrollment Practices

Patients were enrolled according to the eligibility criteria listed below. Enrollment teams attempted to enroll all patients with laboratory-confirmed RSV, SARS-CoV-2, or influenza based on clinical viral testing, if they met syndromic criteria for acute respiratory illness (ARI, see inclusion criteria below). Additionally, for each enrolled patient with confirmed RSV, SARS-CoV-2, or influenza, enrollment teams also enrolled a patient who met syndromic criteria for ARI and either tested negative for RSV, SARS-CoV-2, or influenza based on clinical viral testing or did not receive clinical viral testing for at least one of these viruses.

In addition to clinical viral testing in the local hospital, nasal swabs were collected from enrolled patients and tested for RSV, SARS-CoV-2, and influenza by RT-PCR using standardized methods at Vanderbilt University Medical Center. Patients who originally had negative or no clinical viral testing and subsequently tested positive for RSV, SARS-CoV-2, or influenza on central testing were included as laboratory-confirmed respiratory viral patients for this analysis. Thus, patients included in this analysis were hospitalized with ARI and tested positive for RSV, SARS-CoV-2, or influenza either locally based on clinical test results available at the time of enrollment or centrally in the IVY Network central laboratory.

Patients with laboratory-confirmed RSV, SARS-CoV-2 or influenza based on clinical viral testing at enrollment

Inclusion Criteria:

1. Age ≥18 years old.
2. Hospital admission or in an emergency department awaiting hospital admission.
3. Symptoms and/or signs compatible with an acute viral infection, including at least 1 of the following: fever; cough; shortness of breath; hypoxemia (for patients not on chronic supplemental oxygen, hypoxemia is defined as: SpO2 <92% or use of supplemental oxygen to maintain SpO2 ≥92%; for patients on chronic supplemental oxygen, hypoxemia is defined as SpO2 below the patient's baseline SpO2 or an escalation of supplemental oxygen use to maintain the baseline SpO2 value); new pulmonary findings on chest imaging consistent with pneumonia.
4. Clinically obtained test that is positive for acute RSV, SARS-CoV-2, or influenza after onset of symptoms for the current illness. The positive test may be obtained before or after hospital arrival. Examples of acute viral tests include RT-PCR tests, nucleic acid amplification tests (NAAT), and antigen tests. Serology testing may not be used for eligibility.

Exclusion Criteria:

1. Patient was admitted to the hospital more than 7 days ago (based on this exclusion criterion, patients must be enrolled within 7 days of hospital admission).
2. The first positive test for RSV, SARS-CoV-2, or influenza is known to have occurred more than 10 days after onset of acute viral infection symptoms/signs listed in inclusion criterion #3. Patients with unknown symptoms/signs onset date may be enrolled.
3. First positive test for acute RSV, SARS-CoV-2, or influenza is more than 3 days after hospital admission.
4. Previously enrolled in this surveillance program within the prior 30 days.

© 2024 Surie D et al. *JAMA Network Open.*

Patients with either negative or no clinical viral testing at enrollment

<u>Inclusion Criteria:</u>

1. Age ≥18 years old.
2. Hospital admission or in an emergency department awaiting hospital admission.
3. Symptoms and/or signs that overlap with an acute viral respiratory infection, including at least 1 of the following: fever; cough; shortness of breath; hypoxemia (for patients not on chronic supplemental oxygen, hypoxemia is defined as: SpO2 <92% or use of supplemental oxygen to maintain SpO2 ≥92%; for patients on chronic supplemental oxygen, hypoxemia is defined as SpO2 below the patient's baseline SpO2 or an escalation of supplemental oxygen use to maintain the baseline SpO2 value); new pulmonary findings on chest imaging consistent with pneumonia.
4. Clinically obtained test that is negative for acute RSV, SARS-CoV-2, or influenza after onset of symptoms for the current illness. The negative test may be obtained before or after hospital arrival. Examples of acute viral tests include RT-PCR tests, NAAT, and antigen tests. Serology testing may not be used for eligibility.

<u>Exclusion Criteria:</u>

1. Patient was admitted to the hospital more than 7 days ago (based on this exclusion criterion, patients must be enrolled within 7 days of hospital admission).
2. The first negative test for acute RSV, SARS-CoV-2, or influenza infection is known to have occurred more than 10 days after onset of symptoms/signs listed in inclusion criterion #3. Patients with unknown onset date for symptoms/signs may be enrolled.
3. First negative test for acute viral infection (RSV, SARS-CoV-2, or influenza) more than 3 days after hospital admission.
4. Any positive test for acute RSV, SARS-CoV-2 or influenza infection after symptom onset for the current illness.
5. Previously enrolled in this surveillance program within the prior 30 days.
6. Inability to obtain an upper respiratory sample for central laboratory testing within 10 days of symptom onset for the current illness.

2. Classification of Vaccination Status

Vaccination status for both COVID-19 and influenza were obtained from electronic medical records (EMR), state or jurisdictional registries, and by self-report. Available vaccination data from each of these sources were collected, including date of vaccine administration, vaccine manufacturer, and lot number. Final vaccination status was determined by combining data from verified documented sources (EMR and registry data) as well as plausible self-report based on date of vaccination.

COVID-19 vaccination status

Patients were classified into two COVID-19 vaccination groups: 1) unvaccinated, defined as no prior receipt of COVID-19 vaccination and 2) vaccinated against COVID-19 ≥7 days before illness onset, including receipt of original (ancestral strain) monovalent vaccines (≥2 doses of BNT1262b2 [Pfizer-BioNTech], mRNA-1273 [Moderna], or NVX-CoV2373 [Novavax], or ≥1 dose of Ad26.COV2.S [Janssen]) or ≥1 dose of BNT1262b2 Bivalent vaccine (Pfizer-BioNTech) or mRNA-1273.222 (Moderna)

© 2024 Surie D et al. *JAMA Network Open.*

bivalent vaccine. Patients who received bivalent vaccination may have previously received 1–5 doses of the original (ancestral strain) monovalent vaccines.

Influenza vaccination status

Influenza vaccination status was classified into two groups: 1) unvaccinated, defined as no receipt of influenza vaccine during the current season based on admission date, or 2) vaccinated, if they had received the current season's influenza vaccination ≥14 days before illness onset. Because the period of this analysis spans two influenza seasons, patients enrolled during February 1–July 31, 2022 were classified as vaccinated if they received ≥1 dose of influenza vaccination on or after August 1, 2021 and patients enrolled during August 1, 2022–May 31, 2023 who received ≥1 dose of influenza vaccination on or after August 1, 2022 were also classified as vaccinated.

3. In-Hospital Outcome Definitions

All patients included in this analysis were hospitalized adults aged ≥18 years with symptoms or signs of an acute respiratory illness who tested positive for RSV, SARS-CoV-2, or influenza within 10 days of illness onset and 3 days of hospital admission. Among these hospitalized patients, this analysis compares the association of RSV disease vs. COVID-19 or influenza disease with the following six outcomes that are not mutually exclusive, which are defined in this section:

i. Respiratory virus-associated use of supplemental oxygen therapy
ii. Respiratory virus-associated use of advanced respiratory support
iii. Respiratory virus-associated acute organ failure
iv. Respiratory virus-associated intensive care unit (ICU) admission
v. Respiratory virus-associated hospital-free days
vi. Respiratory virus-associated IMV or death

i. Respiratory virus-associated use of supplemental oxygen therapy during hospitalization:

Patients who met the definition of respiratory virus-associated use of supplemental oxygen therapy during hospitalization either required supplemental oxygen therapy at any time in the hospital prior to day 28 for those not on chronic oxygen or, for patients on chronic supplemental oxygen (**Table**), required an escalation in respiratory support. Oxygen therapy could be delivered at any flow rate and by any device; this included standard-flow supplemental oxygen (flow rate <30 liters/minute), high-flow nasal cannula (HFNC), non-invasive ventilation (NIV), and invasive mechanical ventilation (IMV).

Classification of in-hospital respiratory outcome based on type of oxygen therapy or respiratory support used chronically (before acute illness) and highest level received during the first 28 days of hospitalization.					
Chronic pre-illness oxygen use	Oxygen use during hospital course (highest support)	Is the patient eligible for this outcome?			
		Oxygen therapy	Advanced Respiratory support	Acute organ failure	Invasive mechanical ventilation
No oxygen use	Standard flow oxygen	Yes	No	No	No
	High-flow nasal cannula (HFNC)	Yes	Yes	Yes	No
	NIV	Yes	Yes	Yes	No
	IMV	Yes	Yes	Yes	Yes

© 2024 Surie D et al. *JAMA Network Open.*

Standard flow oxygen	Standard flow oxygen	No	No	No	No
	HFNC	Yes	Yes	Yes	No
	NIV	Yes	Yes	Yes	No
	IMV	Yes	Yes	Yes	Yes
Non-invasive mechanical ventilation (NIV)	Standard flow oxygen	No	No	No	No
	HFNC	No	No	No	No
	NIV	No	No	No	No
	IMV	Yes	Yes	Yes	Yes
Invasive mechanical ventilation (IMV)	Standard flow oxygen	No	No	No	No
	HFNC	No	No	No	No
	NIV	No	No	No	No
	IMV	No	No	No	No

ii. Respiratory virus-associated advanced respiratory support

Patient who meets the definition for advanced respiratory support required use of any of the following during the index hospitalization through hospital day 28: high-flow nasal cannula (HFNC), non-invasive ventilation (NIV), invasive mechanical ventilation (IMV). HFNC was defined as a supplemental oxygen flow rate of at least 30 liters per minute (L/min). NIV included both continuous positive airway pressure (CPAP) and bilevel positive airway pressure (BiPAP) delivered through a mask. A patient was classified as having NIV use in the hospital if NIV was received for therapy of the acute illness and not only for treatment of sleep apnea. IMV was defined as positive pressure administered through an endotracheal tube or tracheostomy tube. Patients who had chronic NIV use before the acute illness met the definition for respiratory virus-associated respiratory support if they had escalation of respiratory support to IMV in the hospital (**Table**). Patients who had chronic IMV use prior to the acute illness were not eligible for the respiratory virus-associated advance respiratory support outcome.

iii. Respiratory virus-associated acute organ failure

Patients were assessed for organ support therapies for the respiratory, cardiovascular, and renal systems during the index hospitalization through hospital day 28. Patients who were newly treated with any of the following organ support therapies met the definition for the acute organ failure outcome:

Organ support for respiratory failure: Patient who meets the definition for respiratory virus-associated hospitalization plus receipt of HFNC, NIV, or IMV during the index hospitalization before day 28. HFNC was defined as a supplemental oxygen flow rate of at least 30 liters per minute. NIV included both continuous positive airway pressure (CPAP) and bilevel positive airway pressure (BiPAP) delivered through a mask. A patient was classified as having NIV use in the hospital if NIV was received for therapy of the acute illness and not only for treatment of sleep apnea. IMV was defined as positive pressure administered through an endotracheal tube or tracheostomy tube. Patients who had chronic NIV use before the acute illness met the definition for respiratory virus-associated respiratory support if they had escalation of respiratory support to IMV in the hospital. Patients who had chronic IMV use prior to the acute illness were not eligible for the respiratory virus-associated respiratory support outcome.

© 2024 Surie D et al. *JAMA Network Open.*

Organ support for cardiovascular failure: Patient who meets the definition for respiratory virus-associated hospitalization plus receipt of intravenous administration of a vasopressor medication by continuous infusion for any duration of time during the index hospitalization before day 28. Vasopressor medications included: norepinephrine, epinephrine, dopamine, phenylephrine, and vasopressin.

Organ support for renal failure: Patient who meets the definition for respiratory virus-associated hospitalization plus receipt of new kidney replacement therapy during the index hospitalization before day 28. Any type of kidney replacement therapy would fulfill the definition for this outcome, including hemodialysis and continuous veno-venous hemofiltration. Patients with chronic kidney replacement therapy prior to the acute illness were not eligible for the influenza-associated kidney replacement therapy outcome.

iv. Respiratory virus-associated intensive care unit (ICU) admission

Patients were classified as having respiratory viral-associated ICU admission if they received care in an ICU for any duration of time during the index hospitalization prior to day 28.

v. Respiratory virus-associated hospital-free days

Hospital-free days to day 28 is a composite of in-hospital death and hospital length of stay defined as the number of days alive and out of the hospital between admission and 28 days later. Patients who died during the hospitalization are classified as having -1 hospital-free days and those who were hospitalized for more than 28 days were classified as having zero hospital-free days. For patients discharged alive before day 28, hospital-free days were calculated as 28 minus the length of stay.

vi. Respiratory virus-associated IMV or death

Patients were classified as having a respiratory virus-associated IMV or death if they met the criteria for IMV (see above) or died in the hospital within 28 days of hospital admission.

4. Laboratory Testing Methods

At the time of participant enrollment, a nasal swab specimen was collected via a fresh swabbing procedure or collection of a residual aliquot in the clinical laboratory. These specimens were frozen at the enrolling site and shipped to Vanderbilt University Medical Center. RT-PCR testing for RSV, SARS-CoV-2, and influenza was completed at Vanderbilt. Specimens with a virus detected were then shipped to the University of Michigan for viral whole genome sequencing. This section describes these laboratory methods.

RSV detection by RT-PCR
For central laboratory pathogen RT-PCR testing at Vanderbilt, total nucleic acid extract from 100 µl of upper respiratory specimen collected in viral transport medium was prepared using the MagNA Pure LC Total Nucleic Acid Isolation Kit (Roche Molecular Systems, Pleasanton, CA) and MagNA Pure 96 automated extraction platform (Roche) or QiaCube HT automated extraction system (Qiagen, Germantown, MD) and QIAamp 96 Virus QiaCube HT kit (Qiagen). Extracts (100 µl eluate volume) were tested by RT-PCR on the StepOnePlus, QuantStudio 3, or QuantStudio 6 Real-Time PCR System (Applied Biosystems, Waltham, MA) for a pan-RSV matrix gene target using Superscript III Platinum One-Step Quantitative RT-PCR System with ROX passive reference dye (Invitrogen, Waltham, MA) and a screening set of primers and probes (forward: GGCAAATATGGAAACATACGTGAA; reverse: TCTTTTTCTAGGACATTGTAYTGAACAG; probe: FAM-CTGTGTATGTGGAGCCTTCGTGAAGCT-BHQ-1) (Biosearch Technologies, Petaluma, CA).

Subgroup differentiation of RSV screen-positive specimens was performed by RT-PCR using Superscript III, a common set of primers, and unique probes targeting A- and B-specific sequences in the viral polymerase (L) gene (forward: AATACAGCCAAATCTAACCAACTTTACA; reverse: GCCAAGGAAGCATGCAATAAA; RSV-A probe: 6FAM-TGCTATTGTGCACTAAAG-MGBNFQ; RSV-B probe: VIC-CACTATTCCTTACTAAAGATGTC-MGBNFQ) (Thermo Fisher, Waltham, MA). Each specimen also was tested for RNase P (RNP) as a marker of specimen adequacy and sensor for PCR inhibitors using TaqPath 1-Step RT-qPCR Master Mix, CG (Applied Biosystems). PCR reactions consisted of 45 amplification cycles, and Ct values of any magnitude were deemed positive when represented by a characteristic specific amplification curve. A valid A or B subgroup identification was contingent on co-detection of the universal RSV target. Absence of RSV detection in specimens registering RNP Ct values ≥40 was considered inconclusive for viral RNA.

RSV RNA extraction and whole genome sequencing
For upper respiratory specimens, RNA was extracted with the MagMAX™ Viral/Pathogen II (MVP II) Nucleic Acid Isolation Kit (Thermofisher), 200 µL of input sample eluted in 50 µL. Extracted RNA was reverse transcribed with the LunaScript® RT SuperMix Kit (NEB). For each sample, 2 µL of mastermix was added to 8 µL of RNA and incubated at 25°C for 2 min, 55°C for 20 min, 95°C for 1 min, and held at 4°C. RSV cDNA was amplified in two multiplex PCR reactions with RSV-A or RSV-B primer pools (**Supplement 2**). Viral cDNA was amplified with the Q5 Hot Start High-Fidelity DNA Polymerase (NEB) with the following thermocycler protocol: 98˚ C for 30 s, then 35 cycles of 95˚ C for 15 s, 63˚ C for 5 min, and final hold at 4˚ C. Reaction products for the two amplicon reactions for a given sample were pooled in equal volumes. Pooled PCR product was purified with 0.8x volume of AMPure beads (BeckmanCoulter). Sequencing libraries were prepared with the NEBNext Ultra II DNA Library Prep Kit (NEB). Barcoded libraries were pooled in equal volume and extracted with a 1% agarose gel to remove adaptor dimers. Pooled libraries were quantified with the Qubit 1X dsDNA HS Assay Kit (Thermo Fisher) and sequenced on an Illumina NextSeq 1000 with a P1 flow cell, 2x300 PE reads, and 384 libraries per flow cell.

Sequencing adaptors and primers were trimmed using cutadapt 1.18. Reads were aligned to reference sequences hRSV/A/England/397/2017 (EPI_ISL_412866) for RSV-A and hRSV/B/Australia/VIC-RCH056/2019 (EPI_ISL_1653999) for RSV-B with BWA-MEM version 0.7.15. Consensus sequences were called with iVar 1.2.1 by simple majority at each position (> 50% frequency), placing an ambiguous N at positions with fewer than 10 reads. Clades were determined using Nextclade. Maximum likelihood phylogenetic trees were generated using IQTree with a GTR model and visualized and annotated using ggtree.

SARS-CoV-2 detection by RT-PCR
For central laboratory pathogen RT-PCR testing at Vanderbilt, total nucleic acid extract from 100 µl of upper respiratory specimen collected in viral transport medium was prepared using the MagNA Pure LC Total Nucleic Acid Isolation Kit (Roche Molecular Systems, Pleasanton, CA) and MagNA Pure 96 automated extraction platform (Roche) or QiaCube HT automated extraction system (Qiagen, Germantown, MD) and QIAamp 96 Virus QiaCube HT kit (Qiagen). Extracts (100 µl eluate volume) were tested by RT-PCR on the StepOnePlus, QuantStudio 3, or QuantStudio 6 Real-Time PCR System (Applied Biosystems, Waltham, MA) for SARS-CoV-2 nucleocapsid (N)-gene N1 and N2 targets and the human RNase P (RNP) gene using the CDC protocol, *CDC 2019-Novel Coronavirus (2019-nCoV) Real-Time RT-PCR Diagnostic Panel* (https://www.fda.gov/media/134922/download). RNP served as an endogenous indicator of specimen adequacy and sensor for PCR inhibitors. The pattern of N1, N2, and RNP Ct values served as a basis to assign a qualitative result of *positive, not detected*, *inconclusive*, or *invalid specimen* with respect to SARS-CoV-2 RNA according to interpretive criteria delineated in the assay protocol.

© 2024 Surie D et al. *JAMA Network Open.*

SARS-CoV-2 RNA extraction and whole genome sequencing

Specimen aliquots with positive RT-qPCR for either the N1 or N2 target with a cycle threshold ≤40 at Vanderbilt University Medical Center laboratory were shipped to the University of Michigan on dry ice. RNA was extracted from 200µl transport media with the Thermo Fisher MagMAX Viral Pathogen II Isolation Kit on a KingFisher instrument and eluted in a 50µl volume. Extracted RNA was reverse transcribed with Lunascript RT Supermix (NEB). For each sample, 2 µl of master-mix was added to 8µl of RNA template and incubated at 25°C for 2 min, 55°C for 20 min, 95C for 1 min. Viral cDNA was amplified in two multiplex PCR reactions with the ARTIC Network (currently version 5.3.2) primer pools and protocol using the Q5 Hot Start High-Fidelity DNA Polymerase Master-mix (NEB) with the following thermocycler protocol: 98˚C for 30 s, then 35 cycles of 95˚C for 15 s, 63˚C for 5 min. Reaction products for a given sample were pooled together in equal volumes. Sequencing libraries were prepared by ligation of the appropriate Illumina or Oxford Nanopore Technologies (ONT) adaptor sets. Three negative control wells (1 HeLa RNA, 2 water) were included on each 96 well RNA harvest plate and carried through the entire process. Barcoded libraries were pooled and sequenced in batches of 96 (GridION instrument) or 384 (Illumina Nextseq 1000, 2x300 P1 flow cell). A run was repeated from RNA harvest on if any of the negative controls have >30x read coverage over 10% of the genome. PANGO lineage was assigned on genomes with >80% coverage using Pangolin v2.4.1 (https://pangolin.cog-uk.io, citation in main text). Genomes with >90% coverage were uploaded to GISAID (https://www.gisaid.org/).

Influenza detection by RT-PCR

For central laboratory pathogen RT-PCR testing at Vanderbilt, total nucleic acid extract from 100 µl of upper respiratory specimen collected in viral transport medium was prepared using the MagNA Pure LC Total Nucleic Acid Isolation Kit (Roche Molecular Systems, Pleasanton, CA) and MagNA Pure 96 automated extraction platform (Roche) or QiaCube HT automated extraction system (Qiagen, Germantown, MD) and QIAamp 96 Virus QiaCube HT kit (Qiagen). Extracts (100 µl eluate volume) were tested by RT-PCR on the StepOnePlus, QuantStudio 3, or QuantStudio 6 Real-Time PCR System (Applied Biosystems, Waltham, MA) for influenza A and B using the CDC Human Influenza Virus Real-Time RT-PCR Diagnostic Panel, Influenza A/B Typing Kit (VER 2). Subtyping and lineage identification of influenza A- and B-positive specimens, respectively, by RT-PCR was performed using the CDC Human Influenza Virus Real-Time RT-PCR Diagnostic Panel, Influenza A Subtyping Kit (VER 3) and CDC Human Influenza Virus Real-Time RT-PCR Diagnostic Panel, Influenza B Lineage Genotyping Kit (VER 1.1). Each specimen also was tested for RNase P (RNP) as a marker of specimen adequacy and sensor for PCR inhibitors using TaqPath 1-Step RT-qPCR Master Mix, CG (Applied Biosystems). PCR reactions consisted of 45 amplification cycles, and Ct values of any magnitude were deemed positive when represented by a characteristic specific amplification curve. A valid influenza A subtype or influenza B lineage identification was contingent on co-detection of the universal influenza type A or B sequence target, respectively. Absence of influenza A and/or B detection in specimens registering RNP Ct values ≥38 was considered inconclusive for the undetected virus(es).

© 2024 Surie D et al. *JAMA Network Open.*

eTable 1. Underlying medical conditions obtained through medical record review.* Condition categories included cardiovascular disease, neurologic disease, pulmonary disease, gastrointestinal disease, endocrine disease, renal disease, hematologic disease, autoimmune disease, and immunocompromising conditions.

**Cardiovascular disease**
Heart failure
Peripheral vascular disease that limits mobility
Prior myocardial infarction
Cardiac arrhythmias including atrial fibrillation and ventricular arrhythmias
Valvular heart disease
Hypertension
**Neurologic disease**
Dementia
Prior stroke
Prior transient ischemic attack (TIA, "mini-stroke")
Brain or spinal cord injury with loss of limb function
Cerebral palsy
Muscular dystrophy
Multiple sclerosis
Myasthenia gravis
Anterolateral sclerosis (ALS)
**Pulmonary disease**
Asthma
Chronic obstructive pulmonary disease
Cystic fibrosis
Pulmonary fibrosis
Pulmonary hypertension
Home oxygen use (except at night for sleep disorder)
Tracheostomy
Home non-invasive ventilation use (except at night for sleep disorder)
Home invasive ventilation use
**Gastrointestinal disease**
Feeding through a tube
Inflammatory bowel disease including Crohn's Disease or Ulcerative Colitis
Cirrhosis (clinical diagnosis of cirrhosis)
Chronic liver disease without cirrhosis
Peptic ulcer disease
**Endocrine disease**
Diabetes mellitus without end organ damage
Diabetes mellitus with end organ damage
Adrenal insufficiency
Hypothyroidism
**Kidney disease**
Chronic kidney disease without chronic kidney replacement therapy
End stage renal disease on chronic kidney replacement therapy (including hemodialysis or peritoneal dialysis)
**Hematologic disease**
Sickle cell disease (all variants)

© 2024 Surie D et al. *JAMA Network Open.*

Coagulopathy or other bleeding disorder, such as hemophilia
Chronic anemia
Thalassemia
**Autoimmune disease**
Systemic Lupus Erythematosus
Rheumatoid arthritis
Psoriasis
Scleroderma
Sarcoidosis
Amyloidosis
Other autoimmune disease
**Immunocompromising conditions**
Active solid tumor or hematologic malignancy (defined as newly diagnosed malignancy or malignancy treatment within the past 6 months)
Solid organ transplant
Hematopoietic cell transplant (HCT)
HIV infection
Primary immunodeficiency
Use of immunosuppressive medication in the past 30 days
Splenectomy
Other conditions that cause moderate or severe immunosuppression

*Obesity is not included in this list of underlying medical conditions.

© 2024 Surie D et al. *JAMA Network Open.*

eTable 2. Characteristics and in-Hospital Outcomes of Adults Aged ≥18 Years Hospitalized with Laboratory-confirmed Coinfections of Respiratory Syncytial virus (RSV), SARS-CoV-2, or Influenza

Characteristic	No. (%) or median (IQR)[a] RSV and SARS-CoV-2 (n = 56)	RSV and influenza (n = 23)	SARS-CoV-2 and Influenza (n = 117)
**Age, median (IQR), y**	64 (48–84)	58 (38–73)	65 (49–76)
**Age, y**			
18–49	15 (26.8)	9 (39.1)	30 (25.6)
50–59	9 (16.1)	3 (13.0)	13 (11.1)
60–69	11 (19.6)	3 (13.0)	31 (26.5)
70–79	16 (28.6)	4 (17.4)	25 (21.4)
≥80	5 (9.8)	4 (17.4)	18 (15.4)
**Race and ethnicity**			
Black, non-Hispanic	11 (19.6)	5 (21.7)	25 (21.4)
Hispanic or Latino, any race	8 (14.3)	6 (26.1)	19 (16.2)
White, non-Hispanic	34 (60.7)	11 (47.8)	58 (49.6)
Other race, non-Hispanic[b]	3 (5.4)	1 (4.4)	11 (9.4)
Other[c]	0 (0.0)	0 (0.0)	4 (3.4)
**Sex**			
Female	30 (53.6)	11 (47.8)	57 (48.7)
Male	26 (46.4)	12 (52.2)	60 (51.3)
**No. of organ systems with chronic medical conditions, median (IQR)[d]**	2 (1–4)	2 (1–2)	2 (1–3)
**In-hospital Outcomes[e]**			
Supplemental oxygen therapy[f]	35 (62.5)	14 (60.9)	78 (66.7)
Advanced respiratory support[g]	9 (16.1)	2 (8.7)	28 (23.9)
Acute organ failure[h]	9 (16.1)	3 (13.0)	30 (25.6)
ICU admission	6 (10.7)	4 (17.4)	18 (15.4)
IMV or death	4 (7.1)	1 (4.4)	16 (13.7)

Abbreviations: IQR = interquartile range; ICU = intensive care unit; IMV = invasive mechanical ventilation; RSV = Respiratory Syncytial Virus

[a] There were a total of 200 laboratory-confirmed respiratory viral coinfections with RSV, SARS-CoV-2, or influenza. Of these, 4 (2%) were triple infections with RSV, SARS-CoV-2, and influenza (data not shown).

[b] Other race, non-Hispanic includes Asian, Native American or Alaska Native, and native Hawaiian or other Pacific Islander which were combined because of small counts.

[c] Other includes patients who self-reported their race and ethnicity as "Other" and those for whom race and ethnicity were unknown.

[d] Organ systems with chronic medical conditions includes: cardiovascular disease, neurologic disease, pulmonary disease, gastrointestinal disease, endocrine disease, kidney disease, hematologic disease, autoimmune disease, and immunocompromising conditions. Details of conditions within each group are in eTable 1 in **Supplement 1**.

[e] Not mutually exclusive.

[f] Supplemental oxygen therapy was defined as use of supplemental oxygen at any flow rate with any device for those not on chronic supplemental oxygen, or as escalation of respiratory support for patients who use chronic supplemental oxygen, at any time during hospitalization prior to day 28.

[g] Advanced respiratory support was defined as receipt of organ support for respiratory failure (high-flow nasal cannula [HFNC], non-invasive ventilation [NIV], or IMV) at any time during the hospitalization before day 28. Patients on chronic home NIV were classified as requiring respiratory support if they received IMV in the hospital. Patients on chronic home IMV were ineligible for this outcome.

[h] Acute organ failure is a composite of respiratory failure (including use of HFNC, NIV, and IMV), cardiovascular failure (use of vasopressors) or renal failure (acute use of kidney replacement therapy).

© 2024 Surie D et al. *JAMA Network Open.*

eTable 3. Components of Composite In-Hospital Outcomes among Adults Aged ≥18 years Hospitalized with Respiratory Syncytial Virus (RSV), COVID-19, or Influenza by Vaccination Status

	No. (%)				
	RSV patients	COVID-19 patients		Influenza patients	
		Unvaccinated	Vaccinated	Unvaccinated	Vaccinated
**In-hospital Outcomes**	**(n = 484)**	**(n = 1422)**	**(n = 5000)**	**(n = 699)**	**(n = 393)**
**Supplemental oxygen therapy**[a]	**355 (73.3)**	**857 (60.3)**	**2924 (58.5)**	**460 (65.8)**	**249 (63.4)**
Standard-flow oxygen	343 (96.6)	798 (93.1)	2785 (95.3)	432 (93.9)	241 (96.8)
High-flow nasal cannula	76 (21.4)	175 (20.4)	479 (16.4)	83 (18.0)	25 (10.0)
Non-invasive ventilation	89 (25.1)	110 (12.8)	356 (12.2)	77 (16.7)	31 (12.5)
IMV	46 (13.0)	166 (19.4)	339 (11.6)	70 (15.2)	18 (7.2)
**Advanced respiratory support**[b]	**146 (30.2)**	**332 (23.3)**	**888 (17.8)**	**157 (22.5)**	**57 (14.5)**
High-flow nasal cannula	76 (52.1)	175 (52.7)	479 (53.9)	83 (52.9)	25 (43.9)
Non-invasive ventilation	89 (61.0)	110 (33.1)	356 (40.1)	77 (49.0)	31 (54.4)
IMV	46 (31.5)	166 (50.0)	339 (38.2)	70 (44.6)	18 (31.6)
**Acute organ failure**[c]	**152 (31.4)**	**359 (25.2)**	**1015 (20.3)**	**170 (24.3)**	**61 (15.5)**
High-flow nasal cannula	76 (50.0)	175 (48.8)	479 (47.2)	83 (48.8)	25 (41.0)
Non-invasive ventilation	89 (58.6)	110 (30.6)	357 (35.2)	77 (45.3)	31 (50.8)
IMV	46 (30.3)	166 (46.2)	339 (33.4)	70 (41.2)	18 (29.5)
Vasopressor use	42 (27.6)	169 (47.1)	415 (40.9)	69 (40.6)	18 (29.5)
Acute use of RRT	7 (4.6)	24 (6.7)	60 (5.9)	4 (2.4)	0 (0.0)
**IMV or death**	**58 (12.0)**	**201 (14.1)**	**458 (9.2)**	**72 (10.3)**	**20 (5.1)**
IMV	46 (79.3)	166 (82.6)	339 (74.0)	70 (97.2)	18 (90.0)
Death	21 (36.2)	84 (41.8)	215 (46.9)	18 (25.0)	6 (30.0)

Abbreviations: IMV = invasive mechanical ventilation; RSV = Respiratory Syncytial Virus; RRT = kidney replacement therapy

[a] Supplemental oxygen therapy was defined as use of supplemental oxygen at any flow rate and by any device for those not on chronic supplemental oxygen, or as escalation of respiratory support for patients who use chronic supplemental oxygen, at any time during hospitalization prior to day 28.
[b] Advanced respiratory support was defined as receipt of organ support for respiratory failure (high-flow nasal cannula (HFNC), non-invasive ventilation (NIV), or IMV) at any time during the hospitalization before day 28. Patients on chronic home NIV were classified as requiring respiratory support if they received IMV in the hospital. Patients on chronic home IMV were ineligible for this outcome.
[c] Acute organ failure is a composite of respiratory failure (including use of HFNC, NIV, and IMV), cardiovascular failure (use of vasopressors) or renal failure (acute use of RRT).

eTable 4. Severity of RSV-Associated Hospitalizations vs. COVID-19-Associated Hospitalizations, by Vaccination Status, Among U.S. Adults Aged ≥60 years

In-hospital outcomes	No. (%) or median (IQR)			RSV vs. COVID-19 by vaccination status			
	RSV patients	COVID-19 patients by vaccination status					
		Unvaccinated	Vaccinated[a]	Unvaccinated		Vaccinated[a]	
	(n = 299)	(n = 745)	(n = 3649)	aOR[b] (95% CI)	*P* value	aOR[b] (95% CI)	*P* value
Supplemental oxygen therapy[c]	238 (79.6)	513 (68.9)	2257 (61.9)	1.89 (1.34–2.65)	<.001	2.44 (1.82–3.28)	<.001
Advanced respiratory support[d]	93 (21.1)	191 (25.6)	649 (17.8)	1.31 (0.96–1.78)	.09	2.11 (1.62–2.75)	<.001
Acute organ failure[e]	97 (32.4)	207 (27.8)	738 (20.2)	1.24 (0.91–1.68)	.17	1.93 (1.49–2.51)	<.001
ICU admission	73 (24.4)	167 (22.4)	591 (16.2)	1.08 (0.77–1.52)	.65	1.60 (1.20–2.14)	.001
Hospital-free days[f], median (IQR)	23 (18–25)	22 (16–25)	23 (19–25)	1.28 (1.01–1.62)[g]	.04	0.81 (0.66–1.00)[g]	.05
IMV or death	40 (13.4)	116 (15.6)	335 (9.2)	0.83 (0.55–1.24)	.36	1.54 (1.07–2.20)	.02

Abbreviations: aOR = adjusted odds ratio; CI = confidence interval; ICU = intensive care unit; IMV = invasive mechanical ventilation; RSV = respiratory syncytial virus.

[a] Includes patients with receipt of the original (ancestral strain) monovalent vaccines, specifically ≥2 doses of BNT1262b2, (Pfizer-BioNTech), mRNA-1273 (Moderna), or NVX-CoV2373 (Novavax), as well as ≥1 dose of Ad26.COV2.S (Janssen) and ≥1 dose of BNT1262b2 Bivalent vaccine (Pfizer-BioNTech) and mRNA-1273.222 (Moderna) bivalent vaccine. Patients who received bivalent vaccination may have previously received 1–5 doses of the original (ancestral strain) monovalent vaccines.

[b] Multivariable logistic regression models were adjusted for age, sex, race and ethnicity, number of organ systems with chronic medical conditions and U.S. Department of Health & Human Services region.

[c] Supplemental oxygen therapy was defined as use of supplemental oxygen at any flow rate with any device for those not on chronic supplemental oxygen, or as escalation of respiratory support for patients who use chronic supplemental oxygen, at any time during hospitalization prior to day 28.

[d] Advanced respiratory support was defined as receipt of organ support for respiratory failure (i.e., acute use of HFNC, NIV, or IMV) at any time during the hospitalization before day 28. Patients on chronic home NIV were classified as requiring respiratory support if they received IMV in the hospital. Patients on chronic home IMV were ineligible for this outcome.

[e] Acute organ failure is a composite of respiratory failure (i.e., acute use of HFNC, NIV, and IMV), cardiovascular failure (i.e., use of vasopressors) or renal failure (i.e., acute use of kidney replacement therapy).

[f] Hospital-free days to day 28 is a composite of in-hospital death and hospital length of stay defined as the number of days alive and out of the hospital between admission and 28 days later. Patients who died during the hospitalization are classified as having -1 hospital-free days and those who were hospitalized for more than 28 days were classified as having zero hospital-free days. For patients discharged alive before day 28, hospital-free days were calculated as 28 minus the length of stay to generate an ordinal scale.

[g] Because hospital-free days is an ordinal outcome, multivariable proportional odds models were used to estimate the association of hospital-free days between RSV and unvaccinated or vaccinated COVID-19. Models were adjusted for the same covariables used in multivariable logistic regression models, including age, sex, race and ethnicity, number of organ systems with chronic medical conditions and U.S. Department of Health & Human Services region.

eTable 5. Severity of RSV-Associated Hospitalizations vs. Influenza-Associated Hospitalizations, by Vaccination Status, Among U.S. Adults Aged ≥60 years

In-hospital outcomes	No. (%) or median (IQR)			RSV vs. Influenza patients by vaccination status			
	RSV patients	Influenza patients by vaccination status					
		Unvaccinated	Vaccinated[a]	Unvaccinated		Vaccinated[a]	
	(n = 299)	(n = 376)	(n = 288)	aOR (95% CI)[b]	*P* value	aOR (95% CI)[b]	*P* value
Supplemental oxygen therapy[c]	238 (79.6)	271 (72.1)	194 (67.4)	1.45 (0.99–2.12)	.06	1.94 (1.30–2.90)	.001
Advanced respiratory support[d]	93 (21.1)	88 (23.4)	39 (13.5)	1.57 (1.09–2.25)	.01	3.06 (1.98–4.73)	<.001
Acute organ failure[e]	97 (32.4)	94 (25.0)	43 (14.9)	1.46 (1.03–2.08)	.04	2.85 (1.87–4.35)	<.001
ICU admission	73 (24.4)	83 (22.1)	30 (10.4)	1.15 (0.78–1.68)	.49	2.70 (1.66–4.41)	<.001
Hospital-free days[f], median (IQR)	23 (18–25)	23 (20–25)	24 (22–26)	0.74 (0.56–0.97)[g]	.03	0.47 (0.35–0.64)[g]	<.001
IMV or death	40 (13.4)	32 (8.5)	15 (5.2)	*[h]		*[h]	

Abbreviations: aOR = adjusted odds ratio; CI = confidence interval; ICU = intensive care unit; IMV = invasive mechanical ventilation; RSV = respiratory syncytial virus.

[a] Patients were classified as vaccinated against influenza if they received seasonal influenza vaccine based on the period in which they were enrolled.

[b] Multivariable logistic regression models were adjusted for age, sex, race and ethnicity, number of organ systems with chronic medical conditions and U.S. Department of Health & Human Services region.

[c] Supplemental oxygen therapy was defined as use of supplemental oxygen at any flow rate with any device for those not on chronic supplemental oxygen, or as escalation of respiratory support for patients who use chronic supplemental oxygen, at any time during hospitalization prior to day 28.

[d] Advanced respiratory support was defined as receipt of organ support for respiratory failure (i.e., acute use of HFNC, NIV, or IMV) at any time during the hospitalization before day 28. Patients on chronic home NIV were classified as requiring respiratory support if they received IMV in the hospital. Patients on chronic home IMV were ineligible for this outcome.

[e] Acute organ failure is a composite of respiratory failure (i.e., acute use of HFNC, NIV, and IMV), cardiovascular failure (i.e., use of vasopressors) or renal failure (i.e., acute use of kidney replacement therapy).

[f] Hospital-free days to day 28 is a composite of in-hospital death and hospital length of stay defined as the number of days alive and out of the hospital between admission and 28 days later. Patients who died during the hospitalization are classified as having -1 hospital-free days and those who were hospitalized for more than 28 days were classified as having zero hospital-free days. For patients discharged alive before day 28, hospital-free days were calculated as 28 minus the length of stay to generate an ordinal scale.

[g] Because hospital-free days is an ordinal scale, multivariable proportional odds models were used to estimate the association of hospital-free days between RSV and unvaccinated or vaccinated COVID-19. Models were adjusted for the same covariables used in multivariable logistic regression models, including age, sex, race and ethnicity, number of organ systems with chronic medical conditions and U.S. Department of Health & Human Services region.

[h] Data not shown as estimates are unreliable because models did not converge.

eFigure 1. RSV Testing among Adults Aged ≥18 Years Hospitalized with Acute Respiratory Illness (ARI)

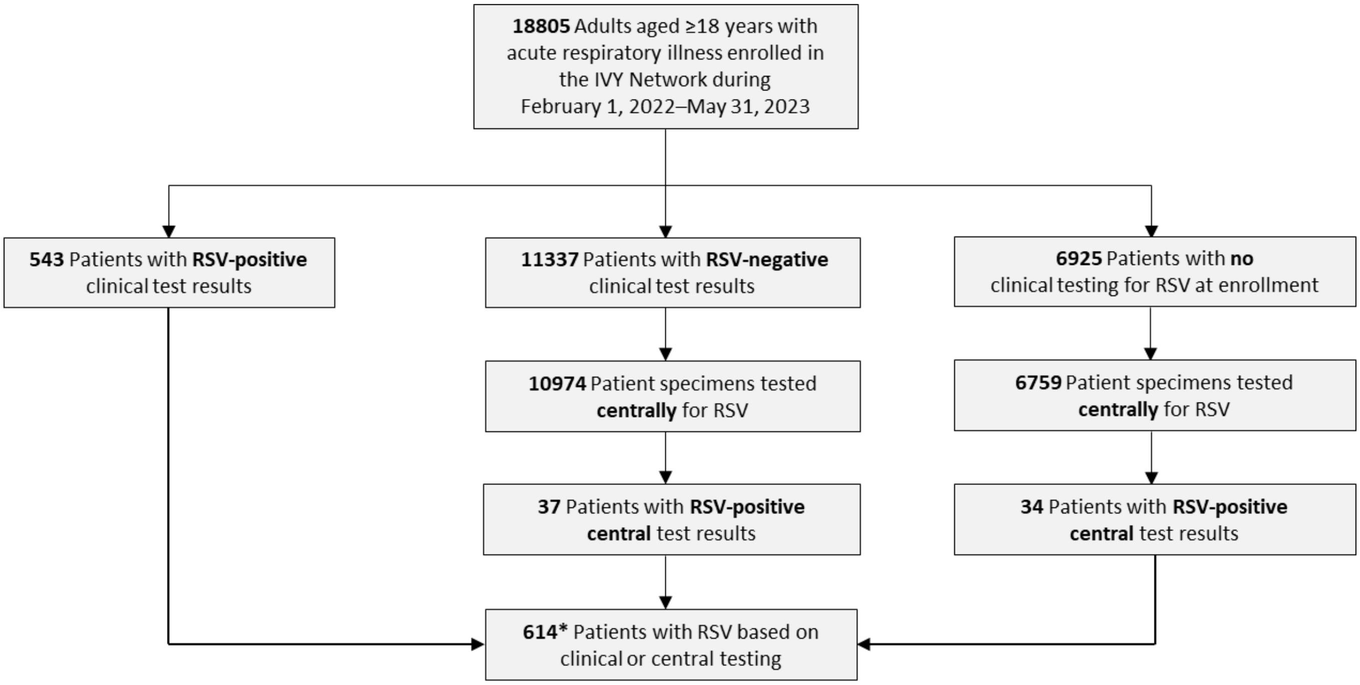

*Includes patients with coinfections; these patients were subsequently excluded from the manuscript analyses (see Supplemental Figure 2)

eFigure 2. Adults Aged ≥18 Years Hospitalized with Respiratory Syncytial Virus (RSV), COVID-19, or Influenza

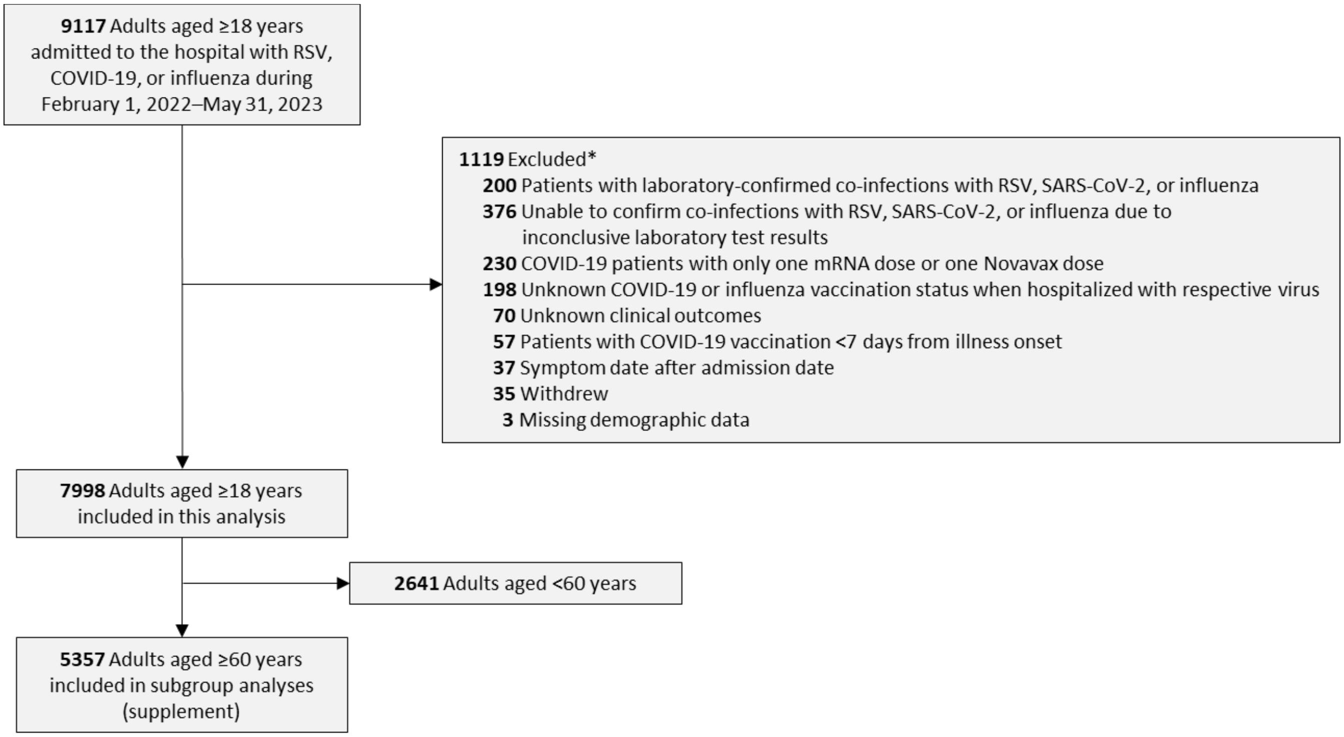

*Not mutually exclusive

eFigure 3. Frequency of Adults Aged ≥18 years Hospitalized with RSV, COVID-19, or Influenza Disease by Admission Week — IVY Network, 25 Hospitals, 20 U.S. States, February 1, 2022–May 31, 2023

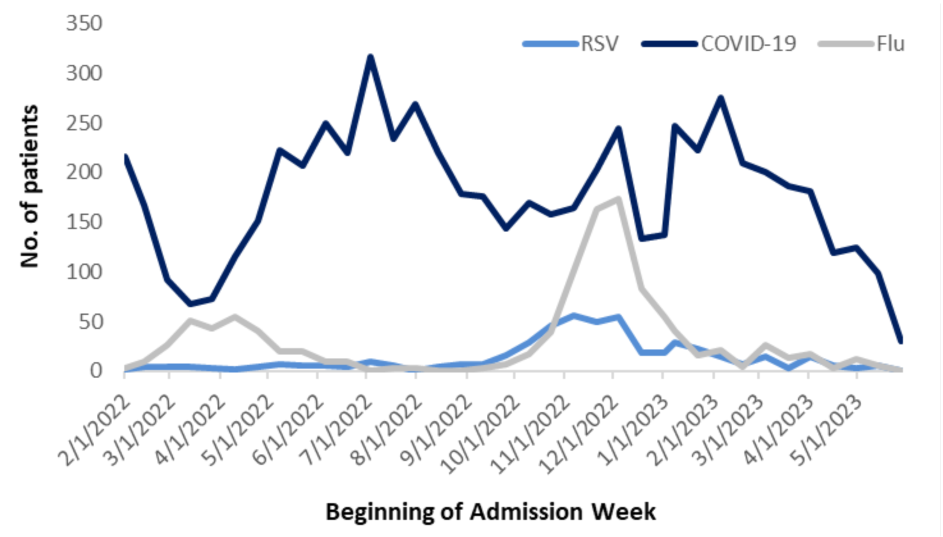

eFigure 4A-C. Frequency of Adults Aged ≥18 years Hospitalized with RSV, COVID-19, or Influenza Disease by Admission Week and U.S. Department of Health and Human Services (HHS) Region — IVY Network, 25 Hospitals, 20 U.S. States, February 1, 2022–May 31, 2023.

A. RSV

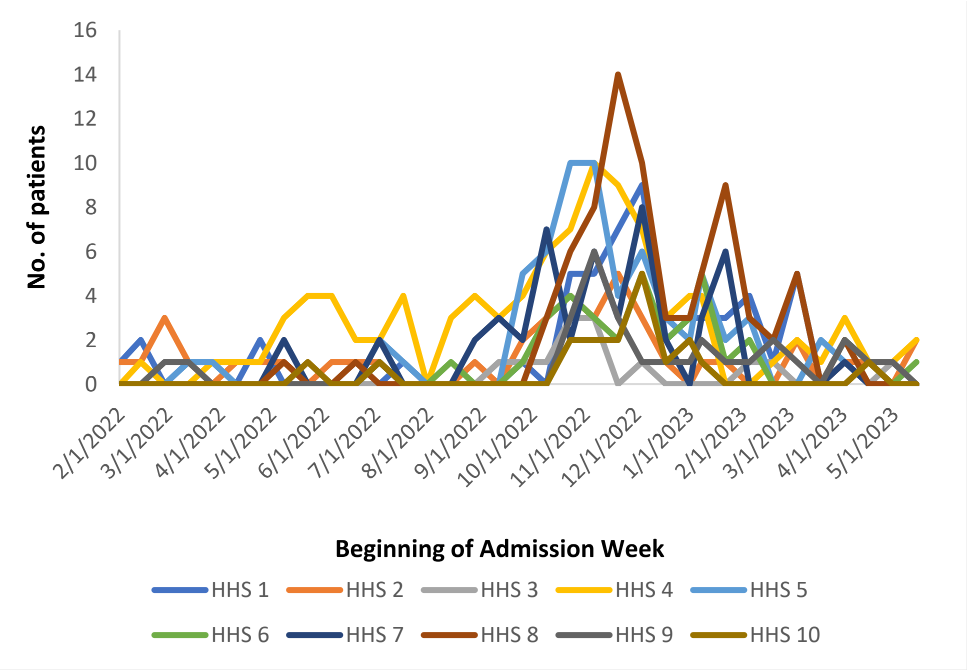

B. COVID-19

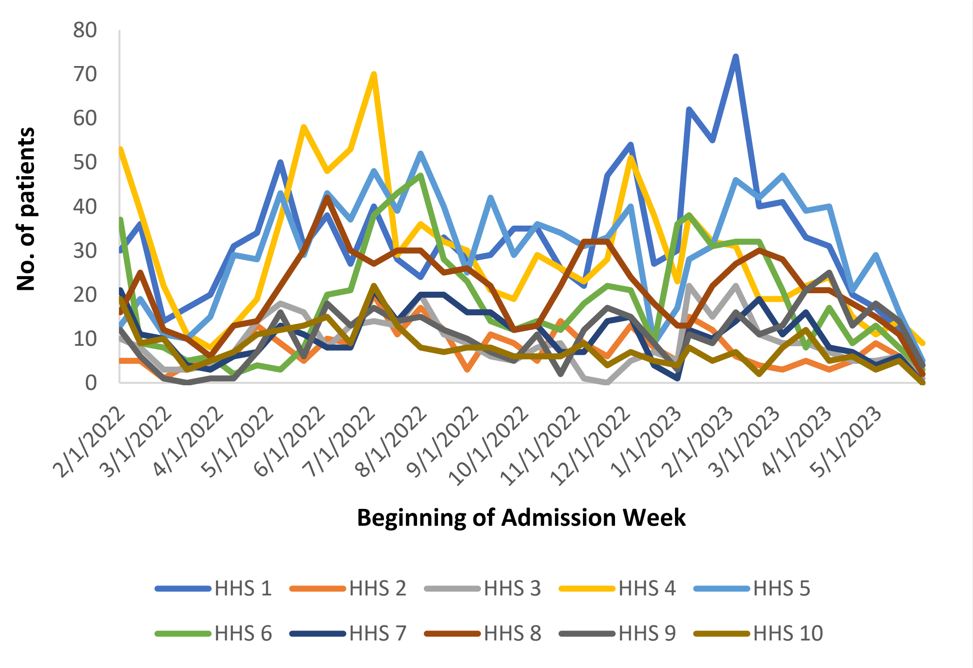

C. Influenza

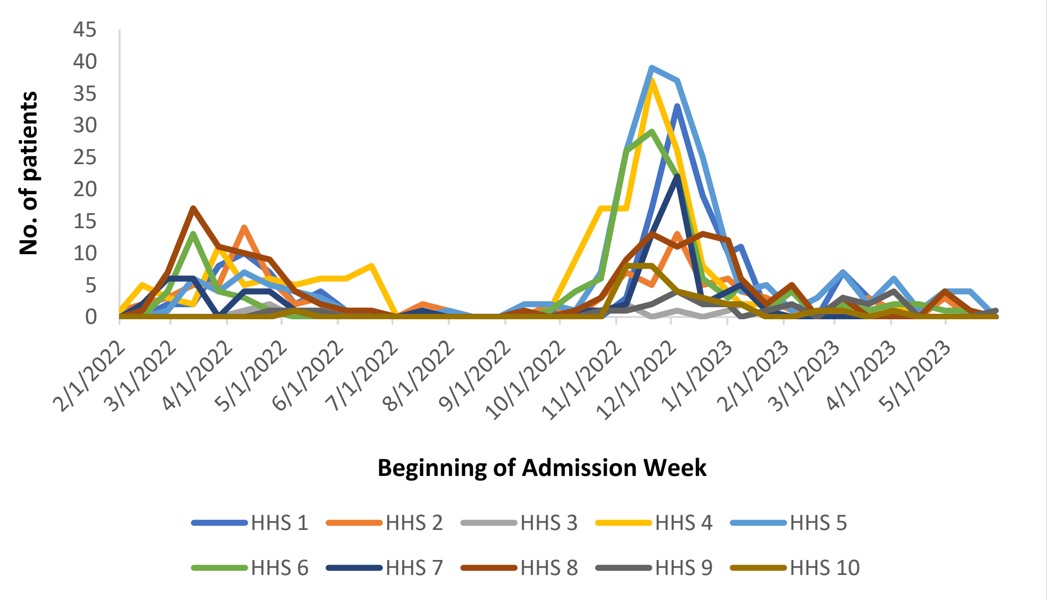

Supplement: Supplement 1. — eAppendix 1. Investigators and Collaborators eMethods. eTable 1. Underlying Medical Conditions Obtained Through Medical Record Review eTable 2. Characteristics and In-Hospital Outcomes of Adults Aged ≥18 Years Hospitalized With Coinfections of RSV, SARS-CoV-2, or Influenza eTable 3. Components of Composite in-Hospital Outcomes Among Adults Aged ≥18 Years Hospitalized With RSV, COVID-19, or Influenza by Vaccination Status eTable 4. Severity of RSV-Associated Hospitalizations vs COVID-19-Associated Hospitalizations, by Vaccination Status, Among US Adults Aged ≥60 Years eTable 5. Severity of RSV-Associated Hospitalizations vs Influenza-Associated Hospitalizations, by Vaccination Status, Among US Adults Aged ≥60 Years eFigure 1. RSV Testing Among Adults Aged ≥18 Years Hospitalized With Acute Respiratory Illness (ARI) eFigure 2. Adults Aged ≥18 Years Hospitalized With RSV, COVID-19, or Influenza eFigure 3. Frequency of Adults Aged ≥18 Years Hospitalized With RSV, COVID-19, or Influenza Disease by Admission Week eFigure 4. Frequency of Adults Aged ≥18 Years Hospitalized With RSV, COVID-19, or Influenza Disease by Admission Week and US Department of Health and Human Services (HHS) Region [file jamanetwopen-e244954-s001.pdf]
