# Supplementary material for: Severity of Respiratory Syncytial Virus vs COVID-19 and Influenza Among Hospitalized US Adults
Source: JAMA Netw Open. 2024 Apr 4;7(4):e244954. doi: 10.1001/jamanetworkopen.2024.4954 (PMC11192181; doi:10.1001/jamanetworkopen.2024.4954)
Supplement: Supplement 4. — Data Sharing Statement [file jamanetwopen-e244954-s004.pdf]

Data Sharing Statement

Surie. Severity of Respiratory Syncytial Virus vs COVID-19 and Influenza Among Hospitalized US Adults. *JAMA Netw Open*. Published April 04, 2024.
doi:10.1001/jamanetworkopen.2024.4954

Data
Data available: No
